# Intrinsic property of phenylalanine to trigger protein aggregation and hemolysis has a direct relevance to phenylketonuria

**DOI:** 10.1038/s41598-017-10911-z

**Published:** 2017-09-11

**Authors:** Bibin G. Anand, Kriti Dubey, Dolat S. Shekhawat, Karunakar Kar

**Affiliations:** 1 School of Life Sciences, Jawaharlal Nehru University, New Delhi, 110067 India; 2 Department of Bioscience and Bioengineering, Indian Institute of Technology Jodhpur, Jodhpur, India 340012

## Abstract

Excess accumulation of phenylalanine is the characteristic of untreated Phenylketonuria (PKU), a well-known genetic abnormality, which triggers several neurological, physical and developmental severities. However, the fundamental mechanism behind the origin of such diverse health problems, particularly the issue of how they are related to the build-up of phenylalanine molecules in the body, is largely unknown. Here, we show cross-seeding ability of phenylalanine fibrils that can effectively initiate an aggregation process in proteins under physiological conditions, converting native protein structures to β-sheet assembly. The resultant fibrils were found to cause severe hemolysis, yielding a plethora of deformed erythrocytes that is highly relevant to phenylketonuria. Unique arrangement of zwitterionic phenylalanine molecules in their amyloid-like higher order entities is predicted to promote both hydrophobic and electrostatic interaction, sufficient enough to trap proteins and to preferentially interact with the membrane components of RBCs. Since the prevalence of hemolysis and amyloid related psychoneurological severities are mostly observed in PKU patients, we propose that the inherent property of phenylalanine fibrils to trigger hemolysis and to induce protein aggregation may have direct relevance to the disease mechanism of PKU.

## Introduction

The multitude of health problems associated with phenylketonuria (PKU) includes disorders such as anemia, rickets, atopic dermatitis, coronary heart disease, diabetes mellitus and arthritis^1–3^ (Supplementary data Table S1). Though several biologically relevant inherent properties of phenylalanine have been reported including its ability to generate β-sheet structured higher order entities and cytotoxic fibrils^4–8^, the question of how these diverse PKU-linked severities arise from a single defect of uncontrolled build-up of phenylalanine in the blood remains largely unanswered. Since PKU symptoms include the occurrence of hemolysis^3^ and the prevalence of amyloid-linked psychoneurological severities such as seizures, hyperactivity and mental retardation^9, 10^, it is very important to understand whether both amyloid fibril formation and hemolysis have any connection with the process of phenylalanine accumulation. We have attempted to gain insight into this fundamental question by testing whether phenylalanine fibrils would drive aggregation of globular proteins that are commonly found in the blood and by exploring what damaging effect such aggregation process would do to the RBCs whose abnormality is highly relevant to PKU.

## Results

### Formation of Phenylalanine fibrils in PBS at 37 °C

**Figure 1:**
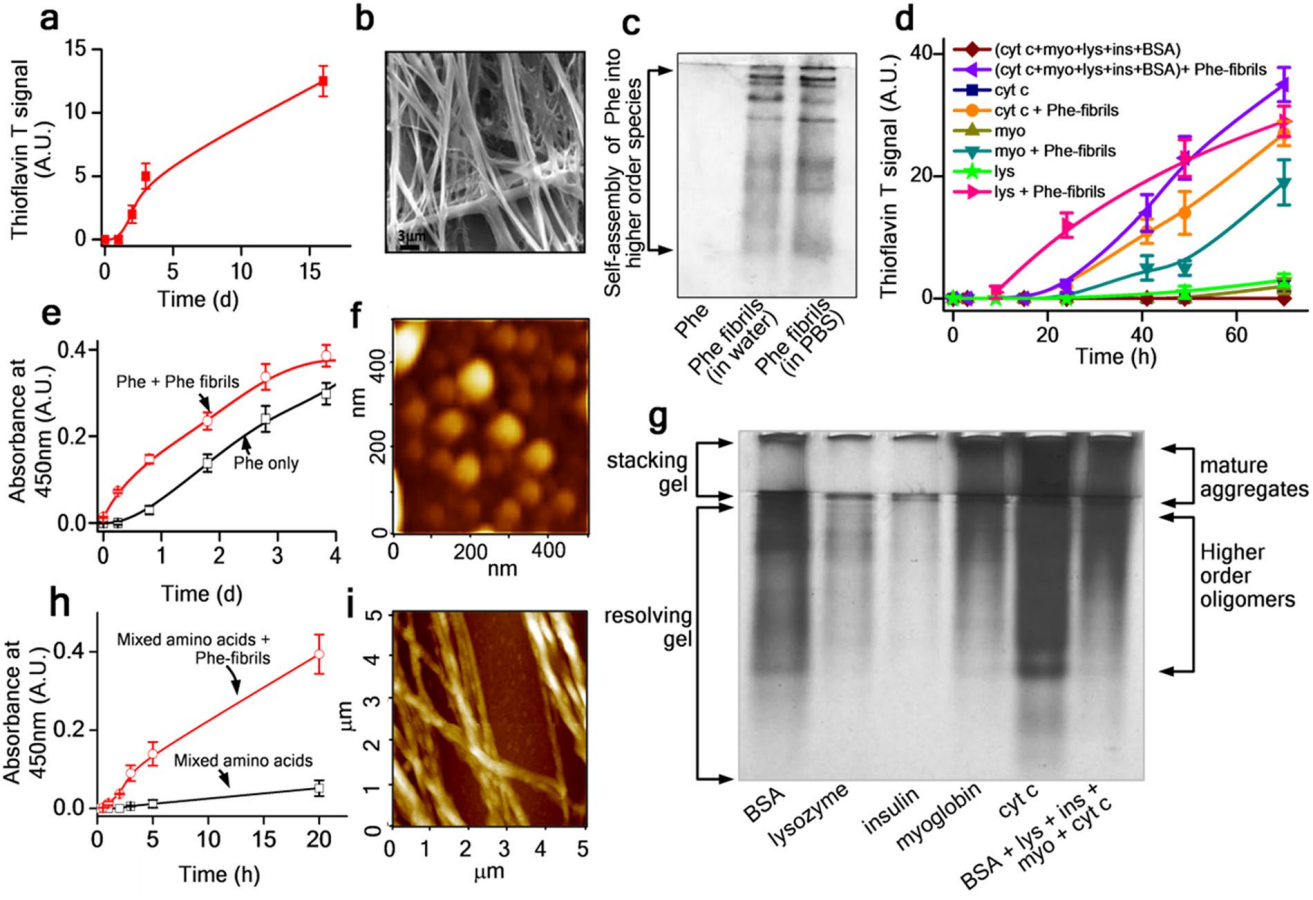
Phenylalanine-fibrils drive aggregation of globular proteins. **(a**) Increase in Thioflavin T signal of 6 mM phenylalanine sample in PBS at pH 7.4. (**b)** Scanning electron microscopy image of phenylalanine-fibrils. Scale bar, 3 μm. (**c**) Self-assembly of phenylalanine into higher order structures, as resolved by Native PAGE. (**d**) Thioflavin T signals revealing rapid aggregation and coaggregation of globular proteins driven by phenylalanine-fibrils as labeled. (**e**) A self-seeded aggregation reaction of phenylalanine. (**f)** AFM image showing spheroidal oligomers of phenylalanine. (**g**) Native PAGE of protein samples confirming aggregation of globular proteins triggered by phenylalanine-fibrils. (**h**) Turbidity data showing aggregation of a soluble mixture of amino acids (Tyr + Trp + Phe + Glu + Arg + Ala), driven by phenylalanine-fibrils. (**i**) AFM images of mature phenylalanine-fibrils.

We generated phenylalanine fibrils by incubating ~6 mM of phenylalanine under physiological conditions of buffer and temperature^4^. The selection of this concentration was based on previous reports that have suggested the amyloid aggregation of Phenylalanine molecules under *in vitro* conditions^4, 11, 12^. Further, it has also been reported that millimolar concentration of phenylalanine can accumulate in the plasma, cerebrospinal fluid and brain tissue^13, 14^. Significant rise in the fluorescence intensity of Thioflavin T, a dye that detects amyloid formation^15^, and increase in the turbidity of the sample were observed (Fig. 1a,e), revealing the conversion of soluble phenylalanine molecules into self-assembled amyloid like higher order structures. The nature of the aggregation reaction appeared to follow a nucleation growth pathway, as no lag time was observed in a self-seeded aggregation reaction (Fig. 1e). SEM and AFM visualisation revealed the formation of both regular fibrils (ranging from ~100 nm to ~3μm) and spheroidal oligomers (~20–80 nm) (Fig. 1b,i,f and Supplementary Data Figs S2, S3 and S4). We confirmed the formation of both low and high molecular weight assembled structures by native PAGE (Fig. 1c).

### Phenylalanine fibrils trigger amyloid formation in proteins in PBS at 37 °C

Because several globular proteins coexist in the body, we questioned what effect phenylalanine fibrils would have on the aggregation propensity of these proteins, particularly those ones that exist in the blood. To address this critical question, we incubated a sample consisting of mixed monomers of selected globular proteins (lysozyme + serum albumin + insulin + myoglobin + cytochrome c) which are commonly found in the blood (Supplementary Table S4). Though the normal level of the protein concentration may vary, due to lysis of RBCs it is much likely that the local concentration of the released proteins may increase to a higher level. All the aggregation reactions were performed in PBS (pH 7.4) at 37 °C to mimic a physiological condition. In the presence of phenylalanine fibrils (~15% w/w), the mixed monomer sample showed an increase in the Thioflavin T signal, suggesting the conversion of soluble monomers of proteins into amyloid like higher order structures (Fig. 1d). To further clarify this seeding effect of phenylalanine-fibrils, we examined its effect on individual proteins and the results indicated a gradual increase in the Thioflavin T signal to a saturation point, confirming the Phe-seed induced spontaneous amyloid fibril formation of individual proteins (Fig. 1d and Supplementary Fig. S1). Without phenylalanine seeds, our control aggregation reactions of both isolated monomers and mixed monomers did not show any indication of aggregation (Fig. 1d). From the preceding results, it is evident that phenylalanine-fibrils can effectively trigger an amyloid aggregation process in proteins under physiological conditions in which proteins usually retain their soluble native conformation. This observation is further supported by previous studies on cross-seeding among proteins or peptides during amyloid formation^16, 17^.

### Structural properties of the protein fibrils

**Figure 2:**
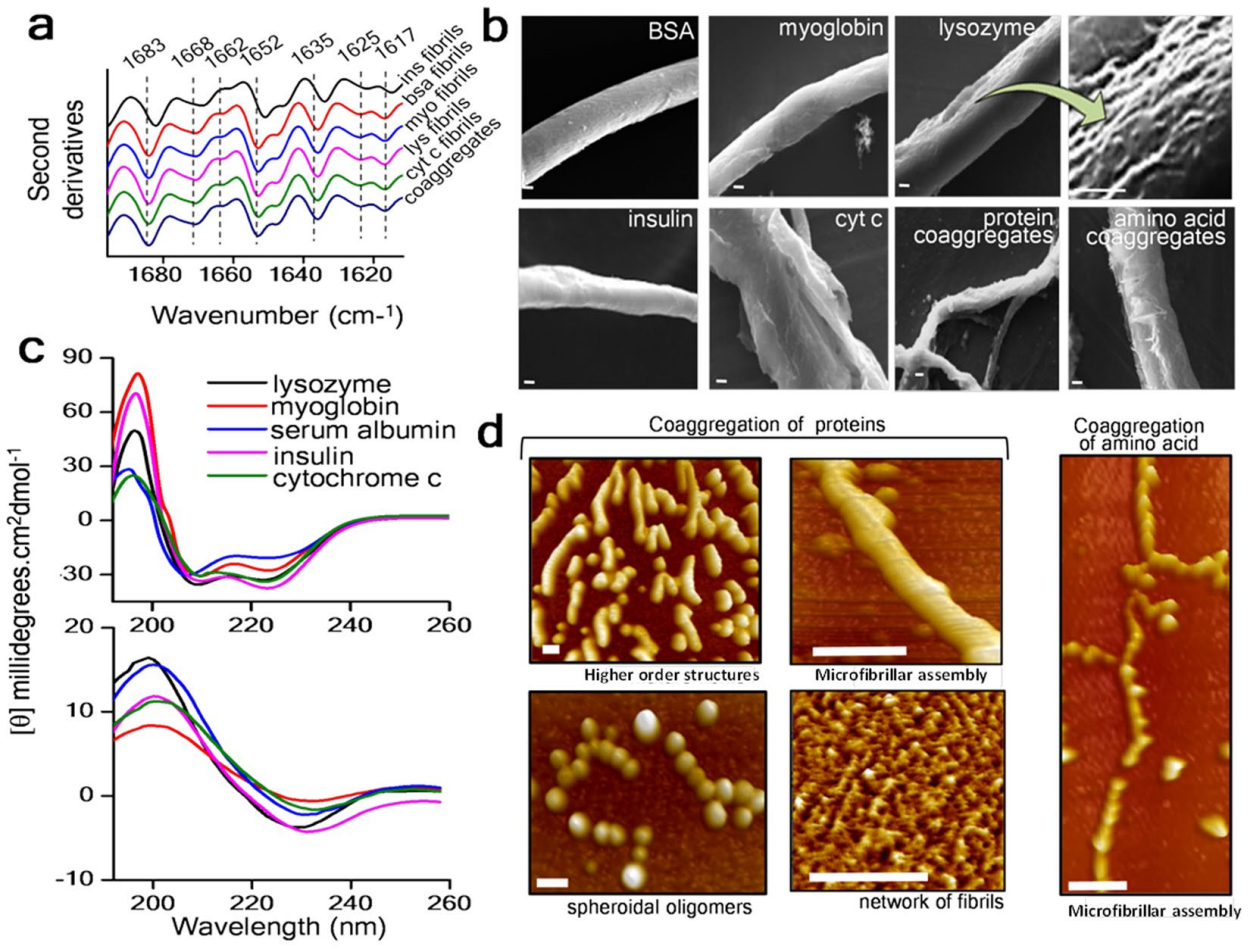
Structural characterization of higher order structures of proteins and amino acids, obtained from phenylalanine-driven aggregation. (**a**) ATR-FTIR spectral signals of mature protein fibrils indicating the dominance of β-structured species, as labelled. (**b**) SEM images of final assembled structure of different proteins. Scale bar, 2 μm. (**c**) Circular dichroism spectra of proteins, showing a conformational switch towards β-sheet structure. (**d**) AFM images revealing higher order structures of proteins and amino acids. Scale bar, 100 nm.

To throw light on this surprising phenylalanine-induced protein aggregation process, we conducted native PAGE experiments which revealed the presence of both mature aggregates and stable oligomers (Fig. 1g). Next, we carried out circular dichroism (CD) measurements and the CD profiles clearly indicated conformational switch towards β-sheet structures (Fig. 2c, Supplementary Table S6). The CD profiles obtained for the protein aggregates do not reflect the typical minima as seen for β-sheet structures, though the pattern of the curves looked similar. We also obtained the CD signal of the sample containing only Phe aggregates in PBS (Supplementary Figure S21) and the peak pattern showed a similar signature as seen for L-Phe sample in solution representing n-π* and π-π* transition^11, 18^. It is also possible that even though β-sheet structures are present in the Phe fibrils, this exciton at 223 nm may be dominating the overall signature of the CD curve. The formation of β-structured fibrillar species after protein aggregation was further confirmed by FTIR data (Fig. 2a). The self-assembly of proteins driven by phenylalanine-fibrils yielded different supramolecular assembled structures including cylindrical microfibrils and twisted flat tapes^19, 20^ (Fig. 2b and Supplementary Data Fig. S5 to Fig. S8), as evident from SEM studies. Notably, the morphologies of the fibrils obtained from aggregation of either mixed monomers or from individual monomers were found to be similar. To further unravel the distinct features of the higher order structures of these proteins, we looked at the AFM images which showed the occurrence of both higher order structures of resembling microfibrils as well as spheroidal oligomers (Fig. 2d and Supplementary Data Figs S9 and S10). Our results obtained from the native PAGE of mature aggregates (Fig. 1g) were found to correlate well with the data obtained from both SEM and AFM experiments (Fig. 2b,d and Supplementary Data Fig. S5 to Fig. S11), particularly revealing the occurrence of both mature fibrils and oligomeric species^21, 22^.

### Coaggregation of amino acids in the presence of Phe fibrils

Since earlier studies have demonstrated the occurrence of spontaneous aggregation of single amino acids under extreme temperature conditions^8, 23^, we wondered whether the phenylalanine-fibrils can drive any such aggregation process of amino acids under physiological conditions. When an aliquot of phenylalanine-fibrils was added to a soluble mixture of amino acids, the sample underwent an aggressive aggregation without any lag phase (Fig. 1h), mimicking a self-seeded aggregation reaction of phenylalanine (Fig. 1e). Examination of these amino acid-generated fibrils by AFM and SEM showed formation of linear fibrils that shared a similar morphology with the protein fibrils (Fig. 2b,d). The resultant fibrils generated from aggregation of amino acids were also found to be Thioflavin T positive, confirming the presence of amyloid like structures (Supplementary Data Fig. S20). Analysis of the nature of the unseeded aggregation reaction of the mixture of amino acids without Phe-seeds (Fig. 1h) also indicates the occurrence of slight aggregation. Since the mixed amino acids sample had phenylalanine as one of the components, it is possible that slight aggregation of this sample may be reflecting the aggregation of the Phe residues. In accordance with such assumption, Phe-fibrils formed during the course of the reaction may also act as seeds to drive aggregation process of other amino acids. To confirm this, we incubated mixture of the same set of amino acids without phenylalanine and no aggregation was observed within the time frame of 24hrs (data not shown). Though Tyr itself has been reported to undergo an aggregation process^12, 18^ under *in vitro* conditions, the concentration requirement was much higher than the concentration maintained in our coaggregation studies. Hence, these data suggest the ability of Phe-fibrils to trigger aggregation reaction in a solution consisting of mixed amino acids.

### Effect of Phe-fibrils and Phe-induced protein fibrils on RBCs

**Figure 3:**
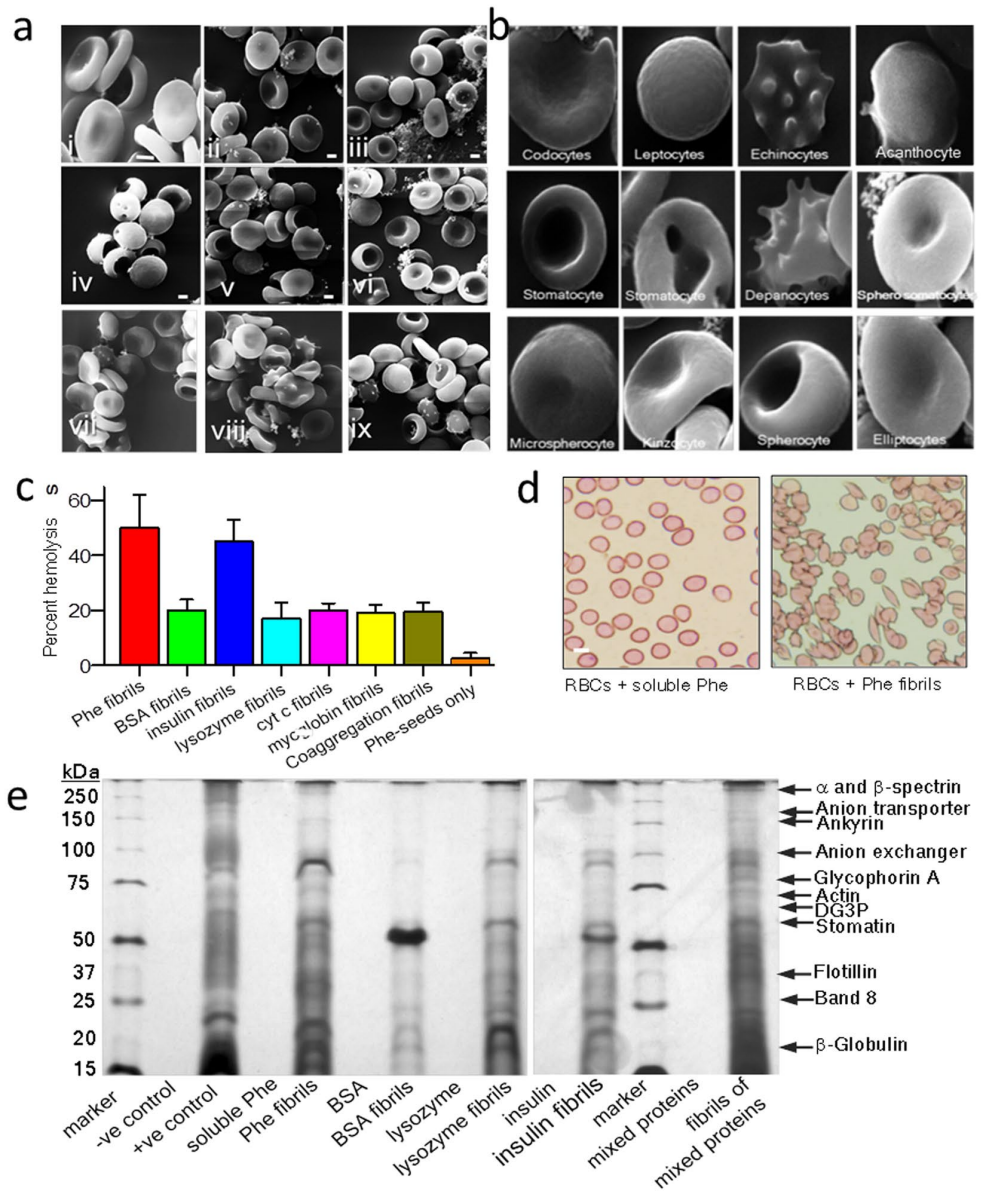
Phenylalanine fibrils and phenylalanine-induced protein fibrils trigger hemolysis. (**a**) SEM images of RBCs in the presence of: (i) 1X PBS, (ii) soluble Phe (iii) Phe-fibrils (iv) BSA fibrils, (v) lysozyme fibrils (vi) insulin fibrils (vii) myoglobin fibrils (viii) cytochrome c fibrils (ix) Coaggregation generated fibrils. All protein fibrils are obtained from phenylalanine fibril-seeded reactions. Scale bar, 3μm. (**b)** Selected SEM images of damaged RBCs revealing the occurrence of pathological deformability, as labelled. (**c)** Histograms showing the percentage hemolysis. (**d**) Optical microscopy images of normal RBCs in the presence of soluble phenylalanine and lysed RBCs in the presence of phenylalanine-fibrils. (**e**) Analysis of lysed erythrocyte membrane proteins as labelled using silver-stained SDS-PAGE.

Having established the inherent ability of the phenylalanine-fibrils to trap soluble globular proteins as well as amino acids into an aggregation pathway that generates β-structured amyloid like higher order entities, we questioned whether these fibrils have any damaging effect on vital blood components, particularly on the RBCs. Since anaemia, a condition caused by lysis of RBCs, is one of the most striking severities found in PKU patients^3^, unravelling the hemolytic effect of these aggregates would be highly relevant to improve our mechanistic understanding of PKU symptoms. Hence, we performed hemolytic assays using RBCs isolated from the blood sample of a healthy volunteer. Phenylalanine, in its soluble state, did not trigger lysis of RBCs, as evident from our SEM (Fig. 3a) and optical microscopy images (Fig. 3d). However, strong lysis of RBCs was observed in the presence of phenylalanine fibrils (Fig. 3a,d). To closely understand the lysis process, we also monitored a time-dependent deformation of intact RBCs in the presence of Phe-fibrils, using dark field microscopy. The deformation of the discoidal shaped normal RBCs was observed within 15–30 mins of time span (Supplementary Data Fig. S14). This result validates the inherent ability of phenylalanine-fibrils to trigger hemolysis which may have direct relevance to the onset of anemia. Interestingly, all the phenylalanine-induced protein aggregates also showed similar hemolytic effect (Fig. 3a, Supplementary Data Fig. S13). SEM revealed a plethora of different deformed RBCs which were identified as acanthocytes, codocytes, leptocytes, echinocytes, echinodacrocytes, microspherocytes, somatocytes, elliptocytes, kinzocytes, spherocytes, epanocytes, and spherosomatocytes (Fig. 3b, Supplementary Data Table S3). The results clearly prove that fibril samples, obtained from both individual aggregation reactions and coaggregation reactions, have similar damaging effect on RBCs. The percentage of lysis (measured considering positive control as100%) caused by phenylalanine-fibrils was observed to be ~50% (Fig. 3c, Supplementary Data Fig. S12), whereas about 20–40% lysis was observed in the presence of different protein aggregates. We also tested the effect of phenylalanine fibrils at a concentration value that was used for seeding the protein aggregation reactions. The data (Fig. 3c) indicated a negligible lysis of ~1.5%, confirming that the toxicity observed in the presence of protein fibrils are not due to the Phe-seeds that were used to induce protein fibril assembly process. To examine the release of different membrane proteins during lysis of RBCs, we performed SDS-PAGE of the lysed RBCs in the presence of both phenylalanine-fibrils and phenylalanine-induced protein fibrils. SDS-PAGE revealed several distinct protein bands (Fig. 3e), confirming the presence of various surface proteins of lysed RBCs^24^.

## Discussion

Our experimental findings provide insight into two inherent properties of phenylalanine that appear highly relevant to PKU: (1) phenylalanine fibrils form a lethal aggregation trap for proteins and amino acids under physiological conditions which yields regular fibrils of amyloid nature; and (2) both phenylalanine fibrils and phenylalanine-induced protein fibrils were found to be highly toxic to RBCs, causing severe lysis. The vast medical significance of deformed RBCs (Supplementary Data Table 3) proposes a direct link between hemolysis and phenylketonuria^3^. Under normal conditions, RBCs maintain a unique biconcave discoidal shape^25^, however the origin of RBC-deformability is clinically attributed to the onset of hemolysis^26^ and sometimes to predispose hypoxia^27^. The damaging effect of amyloid-RBC interaction is known to cause deformability and oxidative reduction in RBCs^27–29^. Interestingly, in this work, in addition to the hemolytic effect a striking tendency of lysed-RBCs to adhere to the surface of phenylalanine-fibrils or proteins-fibrils was observed. Higher hydrophobicity of amyloid fibrils is known to promote its intervention with RBC bilayer^30^. On the other hand, strong interaction between hydrophilic core of phenylalanine oligomers with the cell membrane has been reported to induce cellular damage through ion leakage^6^. The phenylalanine molecules have been reported to form well organized self-assembled tubular like structures^31^ with hydrophobic exterior and hydrophilic interior^32^. Hence, in its amyloid-like higher order entities^4, 6^, phenylalanine is predicted to preferentially interact with the membrane components of RBCs^27, 33^ through viable hydrophobic and electrostatic interactions^34^. Though amyloid aggregation of several globular proteins can be induced through alternations of different solution conditions^23, 35^ such as temperature, concentration and pH which are known to promote formation of aggregation prone partially folded conformations^36, 37^, in this study, it is the native structure that undergoes an aggregation pathway in the presence of phe-fibrils. In their native structures, globular proteins are believed to have solvent exposed charged groups on their surfaces and buried hydrophobic residues within the core of the native conformation. Hence, trapping protein species in their native conformation may be facilitated by the electrostatic interactions between the solvent-exposed charged residues of the protein surface and the charged groups located on the exterior regions of the Phe-fibrils.

In our attempt to study molecular docking Phe with crystal structures of the studied globular proteins, we noticed that Phe has binding affinity for globular proteins (Supplementary Data Fig. S15 to Fig. S19), it is much likely that such crucial phenylalanine-protein interactions would be preferentially favoured when the zwitterionic phenylalanine molecules are arranged within the fibrillar entities^4, 6, 34^. Such crucial contacts may cause the onset of the protein aggregation possibly due to the influence of important aggregation triggering factors: (a) increase in the local concentration of the protein species^38^, (b) a conformational switch in the native structure towards an aggregation-prone state and (c) a preferred intermolecular association between such aggregation-prone species.

Considering the diverse severities linked to Phenylketonuria in which the fundamental defect is accumulation of phenylalanine residues, this work provides enough evidences at least in pointing out two inherent properties of phenylalanine: (a) Phe-fibrils, under physiological conditions drive aggregation of native proteins as well as amino acids; (b) resultant fibrils can cause hemolysis. Occurrence of protein aggregation would lead to deficiency of soluble functional proteins while accumulating toxic amyloid fibrils^39^. Since pathological complications (Supplementary Data Table S4), linked to protein deficiency^40, 41^, amyloid aggregation^35^, and hemolysis^3^ have also been known to occur in PKU, the results of this work probably explains the possible link between a single defect of Phe-accumulation and the occurrence of multiple severities. Though this work has been carried out under *in vitro* conditions, the information gained may perhaps improve our foundational knowledge required for clinical researchers working on cell models and animal models, which may help in the mechanistic understanding as well as in the treatment of Phenylketonuria.

## Materials and Methods

### Reagents

Thioflavin T, bovine serum albumin (BSA) and myoglobin were procured from Sigma-Aldrich. Insulin, lysozyme, cytochrome *c*, phenylalanine, proline, tyrosine, tryptophan, phosphate buffer saline were obtained from Sigma-Aldrich. All other reagents and chemicals used in this work were purchased from Sigma-Aldrich. Protein concentrations were measured by UV 1800 Shimadzu spectrophotometer. Extinction coefficient values used are as follows: 43824 M^−1^cm^−1^ at 280 nm for BSA, 6080 M^−1^cm^−1^ at 278 nm for insulin, 2.6 mg ml^−1^ at 280 nm for lysozyme, 12.8 mM^−1^cm^−1^ at 550 nm for myoglobin and 28 mM^−1^cm^−1^ at 550 nm for cytochrome *c*.

### Study of amyloid fibril assembly

Amyloid aggregation kinetics of proteins was monitored by following ThT fluorescence assay which shows increased fluorescence intensity of ThT as it detects formation of amyloid fibrils during the aggregation reaction. All experiments were performed in an assay buffer (10 mM phosphate buffer saline, pH 7.4 at 37 °C) and concentration of ThT was maintained at 30 μM following established protocol^17, 42^. The ThT fluorescence was recorded as a function of time under ambient conditions, using a Perkin Elmer LS 55 fluorescence spectrometer, with a 440 nm excitation filter and a 495 nm emission filter. Aggregation of phenylalanine monomers (6 mM to 30 mM) was monitored in both water and PBS at room temperature as well as at 37 °C. Aggregation reactions of all the protein samples were carried out at selected concentrations: ~15 μM for BSA, ~174 μM for insulin, ~70 μM for lysozyme, ~57 μM for myoglobin, ~82 μM for cytochrome *c*. For the sample containing a mixture of protein monomers, equimolar concentration of all proteins has been maintained at ~2 μM. Phenylalanine-induced aggregation was carried out in PBS at 37 °C. Preformed phenylalanine fibrils (~15% w/w) were used as seeds for conducting all seed-induced aggregation reactions of the protein samples. Aggregation kinetic of phenylalanine (~6 mM) and a mixture of amino acids (proline, tryptophan, tyrosine, glutamine, alanine and arginine in equimolar concentrations of 0.1 mM) were carried out in PBS (pH 7.4) at 37 °C. Phenylalanine sample and the sample containing mixture of amino acids were incubated in the presence and absence of phenylalanine-fibrils (used as seeds at ~15% w/w). Then turbidity of the samples was monitored at 450 nm at different time points using UV 1800 Shimadzu spectrophotometer.

### Circular dichroism spectroscopy

For the CD experiments, JASCO CD spectrometer (model J-815-150 L) was used. The path length of the cell was 2 mm. The structural changes in the protein samples incubated with phenylalanine aggregates were observed by monitoring the CD scans (between 260 nm–200nm) before and after aggregation, under ambient conditions.

### Atomic force microscopy

We performed conventional AFM measurements in air of the samples deposited on freshly cleaved bare mica using XE-70 Park Systems. All the samples were diluted 5-folds in miliQ water and then 20 μl aliquot of each sample was deposited over the cleaved mica and the samples were then allowed to air dry. Then the samples were washed drop-by-drop by water and were allowed to air dry again. Images were taken immediately using tapping mode (NC-AFM) with a resonance frequency of 300 Hz. All AFM images were captured under ambient condition.

### ATR-FTIR spectroscopy

A Bruker Vertor 70 spectrometer (equipped with silicon carbide source and MCT detector) was used for obtaining FTIR spectra of mature amyloid fibrils. OPUS 6.5 software (Bruker Co., Germany) was used for data processing. All original spectra of amyloid fibrils of different proteins formed in the presence phenylalanine aggregates were processed for baseline correction between 1700 cm^−1^and 1600 cm^−1^ for further analysis.

### Hemolysis assay

The human blood sample was collected from a volunteer donor all methods were carried out in accordance with relevant guidelines and regulations of Ethics Committee of Indian Institute of Technology Jodhpur, India. All the experimental protocols used for this study were approved by Ethics Committee of Indian Institute of technology Jodhpur (Approval letter no IITJ/EC/2016/02-D). Informed consent was obtained from all the subjects involved in this study prior to conducting experiments. Red Blood Cells (RBCs) were separated out from the serum by spinning the blood at 1500 rpm for 10 minutes. The pellets of RBC’s obtained were washed five times with PBS and then diluted. Briefly, from 25% v/v of diluted RBC’s, a 100 μl was added to phenylalanine aggregates and phenylalanine generated protein aggregates (BSA, insulin, lysozyme, myoglobin, cytochrome *c* and mixed-protein monomers). The control samples such as soluble phenylalanine and soluble protein samples were also incubated separately to confirm the lysis ability of phenylalanine driven protein aggregates. All the samples were incubated at 37 °C for four hours and were vortexed slightly for every half an hour. RBC’s incubated with deionized water and PBS was used as positive and negative controls respectively. After incubation, the samples were vortexed again and were centrifuged at 1600 rpm for 10 min. The obtained pellet was saved for microscopic observation. The supernatant that contained the lysate was carefully separated out to check the absorbance (A) values for calculating percentage of lysis following the established protocol. All the spectroscopic measurements for hemolysis assay were recorded using Shimadzu-UV1800 spectrophotometer.

To check the released surface proteins from RBCs, we carried out SDS gel electrophoresis experiments. The lysate sample obtained after the hemolysis assay was centrifuged at 15000 rpm for 20 minutes at 4 °C after which the resultant pellet was washed in sterile PBS buffer at least three times. The final pellet thus obtained was again resuspended in PBS. The protein samples were then mixed with Laemmli buffer (4% sodium dodecyl sulfate [SDS], 20% glycerol, 10% 2-mercaptoethanol, 0.004% bromophenol blue, 0.125 M Tris-HCl). The mixture was heated using boiling water bath for 5 minutes and loaded on a 12.5% polyacrylamide gel and was operated at ambient conditions at ~30 Amp. Silver staining was performed and the stained gels were visualized by Biorad gel documentation unit. The cell pellete containing RBCs were examined initially by Leishman staining procedure for which the RBC were then placed over glass slide and smeared. The smear was first washed with 100% methanol for 30 seconds. The Leishman stain was added to the fixed smear and was left undisturbed for 15 min. Then the slides were washed immediately by water and viewed under Leica microscope. The exact morphology of RBC was confirmed by Scanning Electron Microscopy (SEM). The cell-pellets were washed in PBS and were fixed by incubating with 1% of glutaraldehyde for 2 hours after that the samples are treated with 2% osmium tetroxide for an hour. The cells was then washed thrice using 10% ethanol and dehydrated by incubating with 10%, 20%, 30%, 50%, 70% and 80% ethanol and then desiccated. Thus obtained RBC pellet was sputtered with gold/palladium and viewed under light microscope and under a Carl Zeiss, EVO 18 Scanning electron microscope.

### Molecular interaction studies of phenylalanine with globular proteins

The molecular level interaction between a ligand and a protein can be studied by discovery studio software. The structure of ligand (Phenylalanine) was obtained from PubChem (CID 6140) and was prepared for interaction studies using ‘prepare ligand wizard’ of Discovery studio 4.0 (DS4). X-ray crystal structure of BSA (PDB ID: 4F5S), insulin (PDB ID: 4I5Z), lysozyme (193 L) cytochrome C (1HRC) and Myoglobin (1DWR) was obtained from Protein Data Bank (PDB) and prepared through ‘prepare protein wizard’ of DS4. The structures were initially cleaned by removing water and heteroatoms leaving behind nascent molecules. The pre-processing and protonation were carried out using CHARMm force fields. The ligand and protein were then docked using a blind approach (undefined active site) with 100 conformations to choose and 100 orientations to refine by following CDocker protocol. The simulations were executed in a Dell precision T5610 workstation with 16 processors and 32 GBs of RAM. Visualizations were performed using DS4 for analysis of crystal structure of protein-ligand complex.

### Native PAGE

Native (non- denaturing) polyacrylamide gel electrophoresis was performed in a 12% gel for the protein aggregate samples of BSA, insulin, lysozyme, cytochrome C and myoglobin generated in the presence of phenylalanine. The acrylamide gel was run at a constant voltage of 10 Amp with a mini-PROTEIN II Bio-Rad electrophoresis system using a Tris-HCl polyacrylamide gel at 4 °C. The Phenylalanine higher order structures were visualized in 10% native gel at a constant voltage of 8 Amp. The gels were then developed by silver staining. The stained gels were visualized by Biorad gel documentation unit.

### SEM of amyloid fibrils

The amyloid fibers generated were observed in SEM. The aggregate samples were centrifuged for 10,000 rpm for 10 minutes at 4 °C. The obtained pellet was then resuspended in milliQ deionized water in order to remove the salts. Then the samples were briefly vortexed thrice and the washed five times by centrifugal separation. Then obtained pellet was resuspended in water and then casted over silver stubs and was sputtered with gold/ palladium for 180 seconds. Thus the prepared samples were imaged in SEM at a constant voltage of 20 kV.

### Data analysis and statistics

Error bars are standard deviations from analyses in either duplicate or triplicate. Data points were fit in Origin v2015 software (Origin Lab). Aggregation data were fit to B-spline curves. Hemolysis data, FTIR and turbidity data were analyzed by OPUS 6.5 software (Bruker Co., Germany). Significance was determined using Student’s t-tests, considering P < 0.05. AFM data were processed using XEI software.

## Electronic supplementary material

Supplementary information
