# Supplementary material for: Intrinsic property of phenylalanine to trigger protein aggregation and hemolysis has a direct relevance to phenylketonuria
Source: Sci Rep. 2017 Sep 11;7:11146. doi: 10.1038/s41598-017-10911-z (PMC5593866; doi:10.1038/s41598-017-10911-z)
Supplement: Supplementary file 1 — Supplementary information [file 41598_2017_10911_MOESM1_ESM.pdf]

Intrinsic property of phenylalanine to trigger protein aggregation and hemolysis has a direct relevance to phenylketonuria.

Bibin G. Anand [¶,§], Kriti Dubey [¶,§], Dolat S. Shekhawat [¶], Karunakar Kar [†*]

Author affiliations:

[†]School of Life Sciences, Jawaharlal Nehru University, New Delhi-110067, India

[¶]Department of Bioscience and Bioengineering, Indian Institute of Technology Jodhpur, India-340012.

[*] Corresponding author: Karunakar Kar; School of Life Sciences, Jawaharlal Nehru University, New Delhi-110067, India; phone: +91-1126704517; email: karunakarkar@gmail.com, kkar@mail.jnu.ac.in

Supplementary Information

Table S1. List of several pathophysiological conditions linked to phenylketonuria with detailed metabolic alterations.

Pathophysiology linked to PKU	Metabolic alterations
Cerebral deficiency	Reduce dopamine and serotonin concentration in blood[1,2]
Neuronal dysfunction and *dementia*	Reduced dopaminergic and serotonergic metabolites in CSF as well as decreased catecholamine and serotonin concentrations in the brain.[3,4]
Oxidative stress, lipid and protein oxidative damage	Deficiency of L-carnitine.[5]
CNS lesions with demylination	Elevated S-100B protein in serum.[6]
Coronary Heart disease, neurological disorder	Low serum cholesterol, HDL,LDL and high

	triglycerides and VLDV.[7,8,14]
Increase adiposity	Elevation of leptin plasma levels.[9]
Mutation	DNA damage in leukocytes.[10,15]
Neurotoxicity	Reduced acetylcholinesterase activity in erythrocyte membranes.[11]
Neurotoxicity, uremia, *rheumatoid arthritis*, *diabetes mellituse*, essential hypertension, spongiform encephalopathy.	Reduced the activities of Na,K-ATPase and Mg2-ATPase in erythrocyte membranes.[12]
Enhance oxidation stress, Lipid oxidation	Ubiquinone 10 (Q10) deficiency in lymphocytes.[13]
Interfere with cholesterol biosynthetic pathway	Inhibition of enzyme 3-hydroxy-3-methylglutaryl CoA reductase.

Table S2. List of selected diseases induced by peptide sequences that containing aromatic residues including phenylalanine.

**Name of parent peptide/protein**	**Pathophysiological conditions**	**Short active Sequence***
Islet amyloid polypeptide	Diabetes mellitus (type II diabetes)	FGAIL[16]
β-Amyloid peptide	Alzhemier's disease	QKLVFF,LPFFD, LVFFA.[17, 18, 19, 20]
Lactadherin	Aortic medial amyloid	NFGSVQFV[21]
Gelsolin	Finnish hereditary amyloidosis	SFNNGDCCFILD[22]
Serum amyloid A	Chronic inflammation amyloidosis	SFFSFLGEAFD[23]
Thyroid carcinoma peptide	Thyroid carcinoma	DFNKF, DFNK[24]
Human muscle acylphosphatase amyloid	Human muscle acylphosphatase	RVQGVCFRMTEDEAR SKLEYSNFSIRY[25]
Calcitonin	Medullary thyroid carcinoma	YTQDFNKFFHTFPPQTAIGV[26, 27, 28]
BRI	Neuronal dysfunction and dementia	FENKFAV FAIRHF[29]
β2-microglobulin	Dialysis-associated renal amyloidosis	DWSFYLLYTEFT[30]
PrP	Creutzfeldt-Jakob disease	PHGGGWGQ[31, 32]

*Phenylalanine is indicated in red and other amromatic residues are represented in green color.

Table S3. List of diseases related to deformability of RBC's.[33, 34]

**Shapes of Erythrocytes**	**Linked Pathophysiology**
Acanthocytes	Abetalipoproteinemia cirrhosis and rarely other liver diseases
Codocytes	Thalassemia
Leptocytes	Hypochromic anemia
Echinocytes	Uremia, Congenital anemia
Echinodacrocytes	Thalassemia, hemolytic anemia with Heniz bodies
Microspherocytes	hereditary Spherocytosis and somatocytosis
Somatocytes	Heriditary or acquired hemolytic anemia
Elliptocytes	Congenital elliptocytosis
Kinzocytes	Hemolytic anemia and hereditary Spherocytosis
Spherocytes	hereditary Spherocytosis, burns and some hemolytic anemia
Depanocytes	Sickle celled anemia
Sphero somatocytes	hereditary Spherocytosis and somatocytosis

Table S4. Physiological concentration of the blood proteins, their functions and related pathophysiology.

Protein	About protein	Function	Protein pathophysiology	Relevance to PKU
BSA	Most abundant serum protein, MW-66.5 kDa, Synthesis site liver (12-25gm/day), Half life – 16 hrs, Reference range in body -3.5 to 5.5 g/dL[53]	Blood coagulation, transports hormones, fatty acids, drugs etc., Maintains oncotic pressure, prevents photodegradation of folic acid	Peripheral and pulmonary oedema delayed wound healing [52] Aephrotic syndrome, hepatic cirrhosis, heart failure, and malnutrition[56,57]	Coronary Heart disease, malnutrition
Insulin	Synthesized in the pancreas within the β-cells of the islets of Langerhans. MW-5.8 kDa, half life 4-5 hrs Reference range in body – Fasting- < 25 mlU/L 30 minutes after glucose -30-230 mlU/L 1hrs after glucose -18-276 mlU/L 2hrs after glucose -16-166 mlU/L 3hrs or more after glucose -‹ 25 mlU/L[54]	Carbohydrate and fat metabolism, facilitates the packing of glucose into fat cells as triglycerides,	Localized amyloid deposition[35], poor glycemic control, acanthosis nigricans[36], hyperinsulinemia is directly related to Cancer[38,39], Ogesity[41], type-II diabetes[43], Hypertension[37], Arthrosclerosis[45], Chronic inflammation, cardio vascular disease[40,41,42,43], ,prostate inlargment[44] etc.	Type-II diabetes, cardio vascular disease, amyloid deposition
Cyt c	Located in the mitochondrial intermembrane. MW- 12kDa, half life 5 to 8 minutes	Electron transport, intrinsic type II apoptosis, scavenges reactive oxygen species, oxidizes cardiolipin during apoptosis	Lewy bodies and other neurodegenerative disorders[51]	Neurodegenerative disorders
Lysozyme	synthesis in osteoclasts MW- 14.3 kDa, half life 4hrs Reference rang in body – In serum -7-13 mg/l In tears about 120 times higher than in serum In urine about 8 time more than in serum[55]	Hydrolyzing the glycosidic bond, Immune functions.	Prominent amyloid nephropathy, nephrotic syndrome and sicca syndrome[46], spontaneaous splenic rupture, cholestasis, and liver failure[47], massive hepatic hemorrhage[48]	
Myoglobin	Synthesis in cardiac myocytes and oxidative skeletal muscle fibers. MW 16.7 kDa, half life	Oxygen storage, serve as a buffer of intracellular PO_2, Facilitated O2 diffusion, scavenger of NO in heart, normal muscle development and function	Muscular dystrophy, rhabdomyolysis[49,50]	

Table S5. Percentage hemolysis of RBC's in the presence of different soluble and aggregated samples of proteins.

**Samples**	**Concentrations**	**% lysis of RBC's, soluble sample**	**% lysis of RBC's, aggregated sample**
Phenylalanine	~6mM	0	53.0
BSA	~13µM	0	20.0
Insulin	~156µM	0	45.0
Lysozyme	~63µM	0	16.5
Cytochrome *c*	~74µM	0	19.9
Myoglobin	~51µM	0	18.8
Co-aggregates	~1.8µM	0	19.0

Table S6. Predicted secondary structures of protein samples before and after aggregation using circular dichroism data**.** K2D3 online tool[58] was used to theoretically predicting the secondary structures of protein samples.

**Samples**	**Soluble Proteins**		**Aggregated Proteins**	
	**α-helices (%)**	**β-sheets (%)**	**α-helices (%)**	**β-sheets (%)**
BSA	67.86	8.5	1.75	33.63
Lysozyme	35.16	17.82	1.09	42.43
Insulin	33.37	18.7	2.78	32.8
Myoglobin	85.64	0.38	2.9	42.7
Cytochrome *c*	78.62	0.29	2.5	41.57

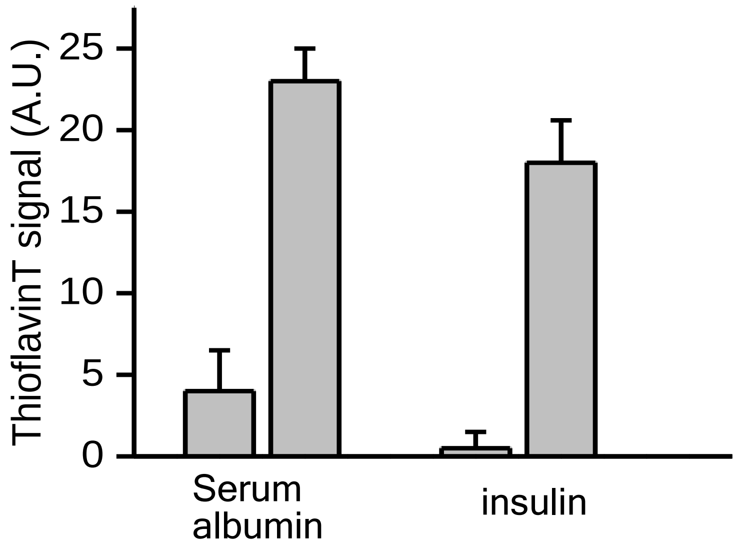

Figure S1. Histogram showing Thioflavin T signals of insulin and BSA at 0 h and 24 h.

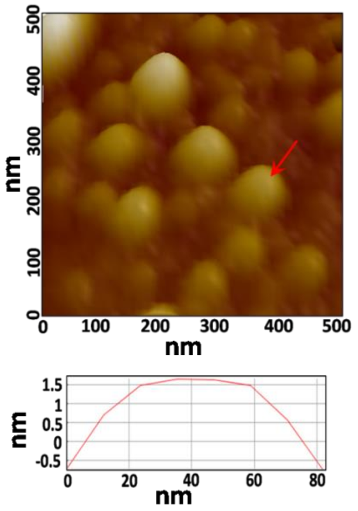

Figure S2. AFM image representing spheroidal oligomers of phenylalanine sample aggregated in water at 37ºC. The diameters range was from ~25 nm-80 nm.

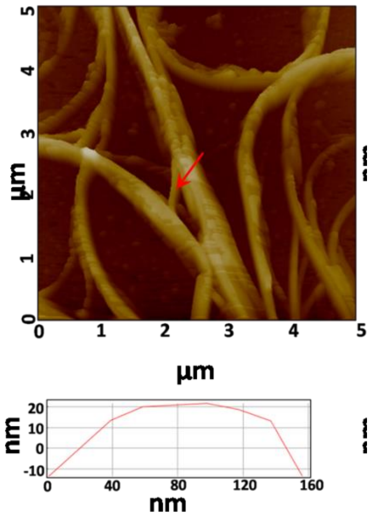

Figure S3. AFM image, representing regular fibrils of phenylalanine sample aggregated in water at 37ºC. The diameter range was between ~100nm-160nm.

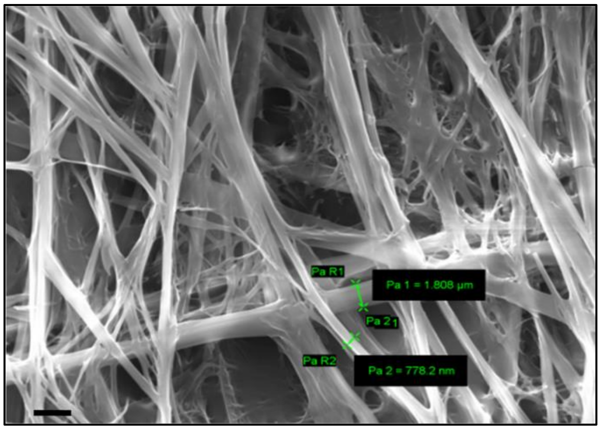

Figure S4. SEM image, representing regular fibrils of phenylalanine sample aggregated in water at 37ºC. Scale bar, ~2 µm.

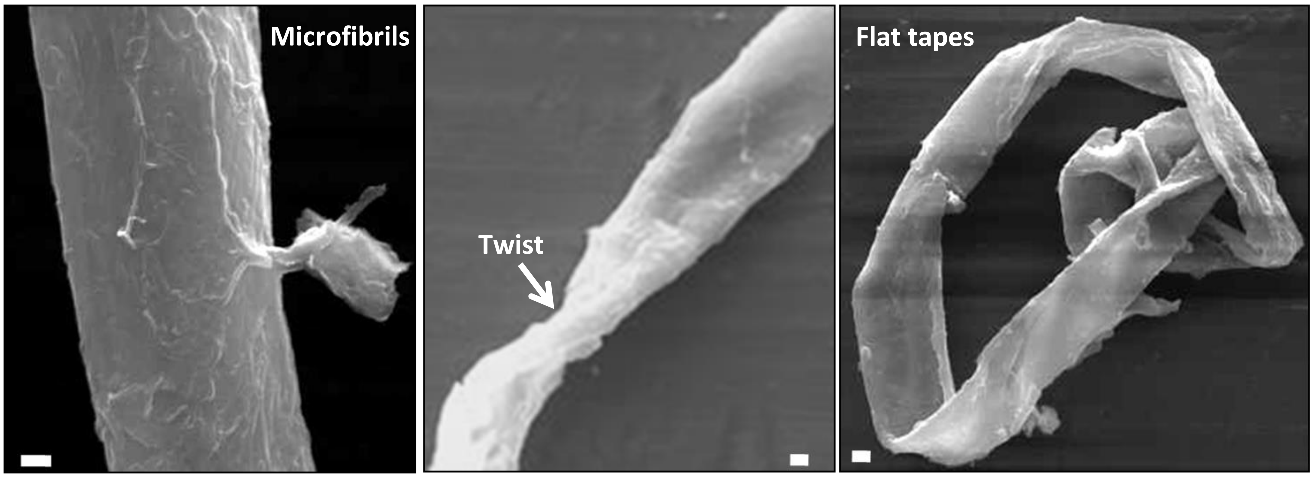

Figure S5. SEM images representing different morphologies of BSA aggregates. Scale bar, ~2 µm.

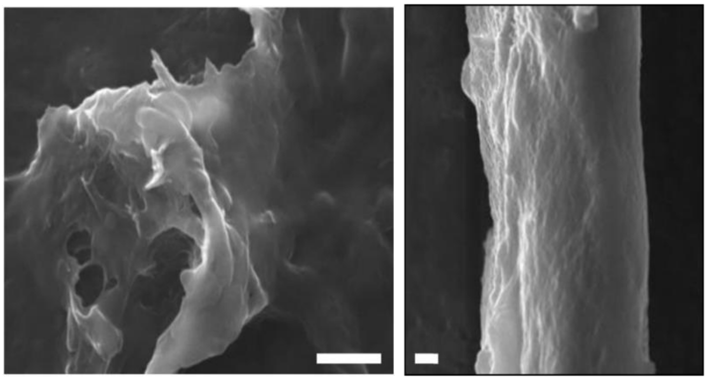

Figure S6. SEM images representing different morphologies of lysozyme aggregates. Scale bar, ~2 µm.

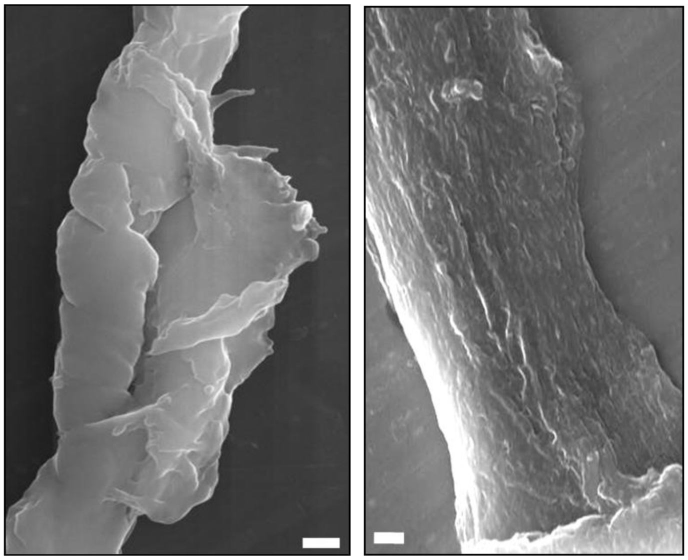

Figure S7. SEM images representing different morphologies of myoglobin aggregates. Scale bar, ~2 μm.

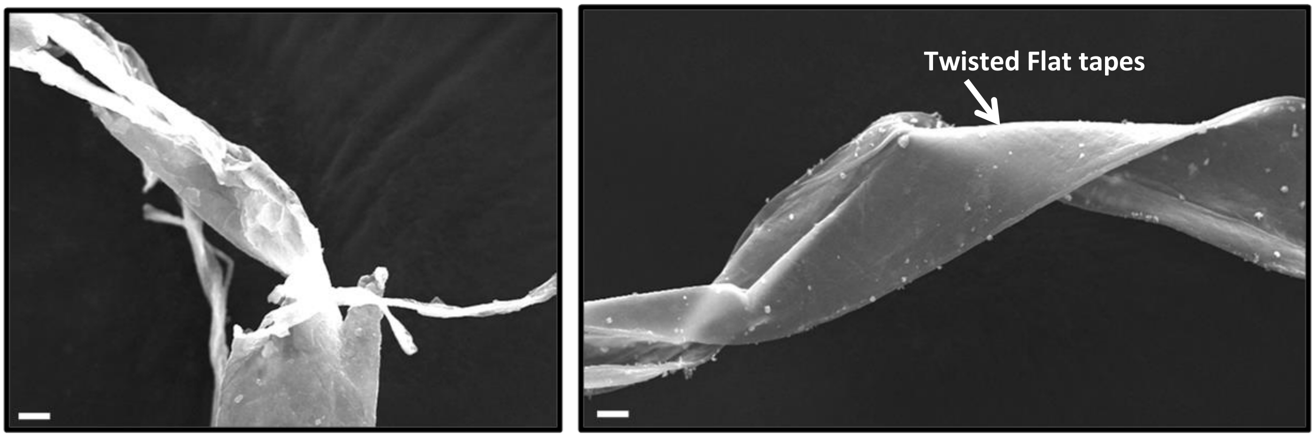

Figure S8. SEM images representing different morphologies of insulin aggregates. Scale bar, ~2 μm.

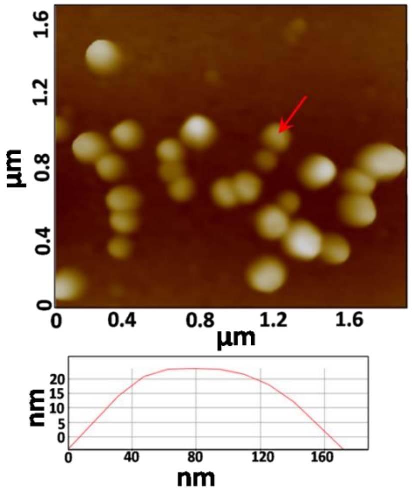

Figure S9. AFM image, representing spheroidal oligomers (within 150-200 nm) observed in phenylalanine induced aggregation of mixed protein monomers (in PBS at 37ºC).

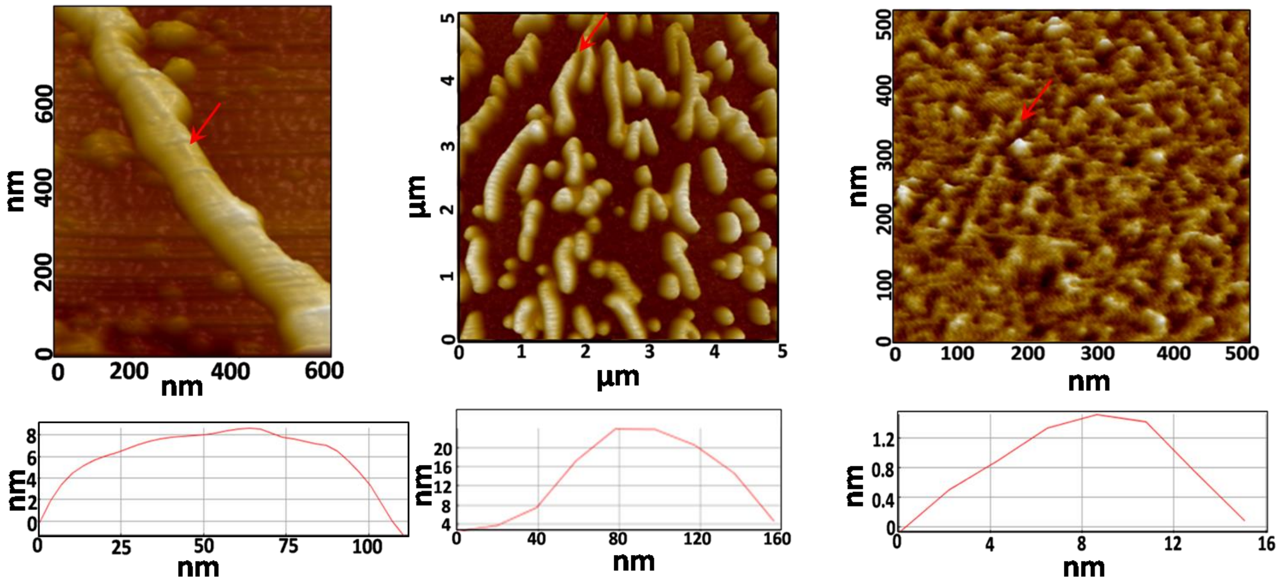

Figure S10. AFM images, representing microfibrils (within 150-200 nm) observed uring phenylalanine induced aggregation of mixed protein monomers (in PBS at 37ºC).

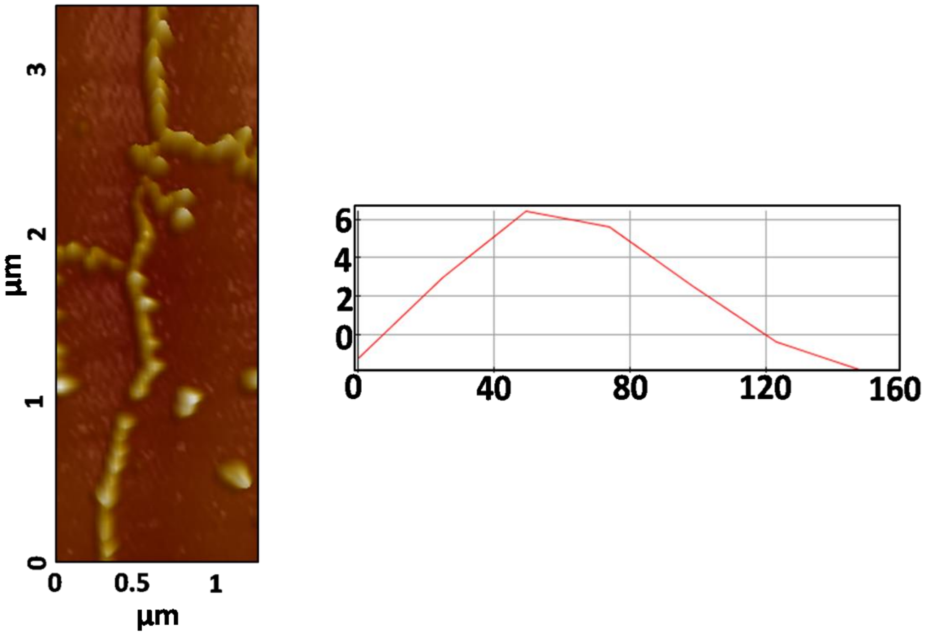

Figure S11. AFM image of higher order structures obtained for phenylalanine-induced aggregation of mixed amino-acids (in PBS, 37ºC).

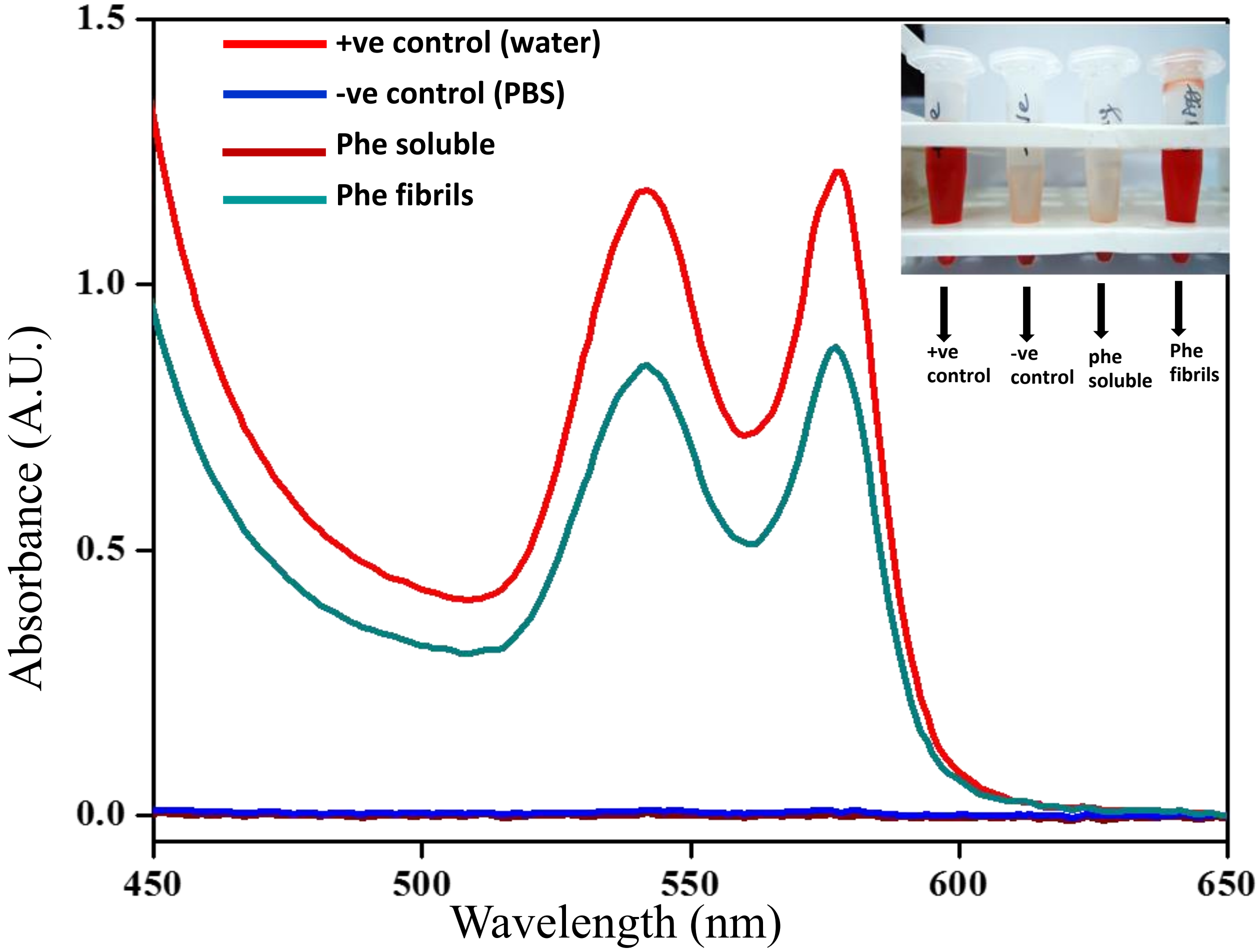

Figure S12. UV absorption spectra obtained for the hemolysis experiment after four hours of incubation at 37°C. Severe lysis was observed in the presence of water (+ve control) and phenylalanine fibrils whereas no lysis was detected in the presence of soluble phenylalanine and in PBS buffer (used as –ve control).

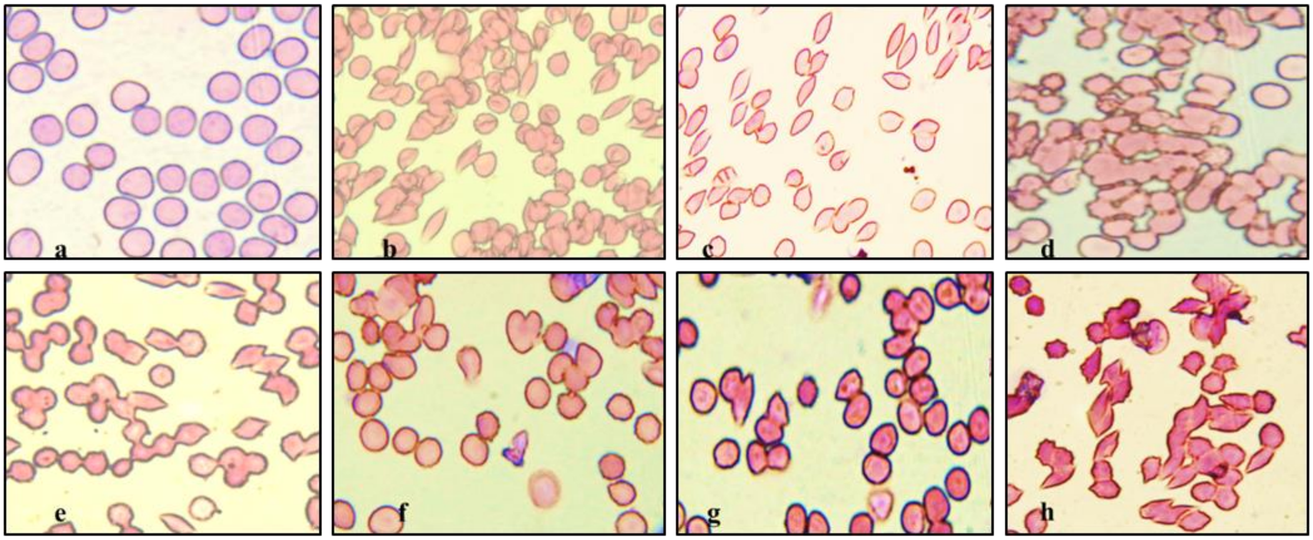

Figure S13. Leishman stained images of RBC's, visualized by the light microscope (Leica DM 500 Switzerland), in the presence of: a) PBS (control RBC's) b) Phenylalanine aggregates c) BSA aggregates d) Insulin aggregates f) Lysozyme aggregates g) Cytochrome C aggregates h) Myoglobin aggregates I) aggregates of mixed protein monomers.

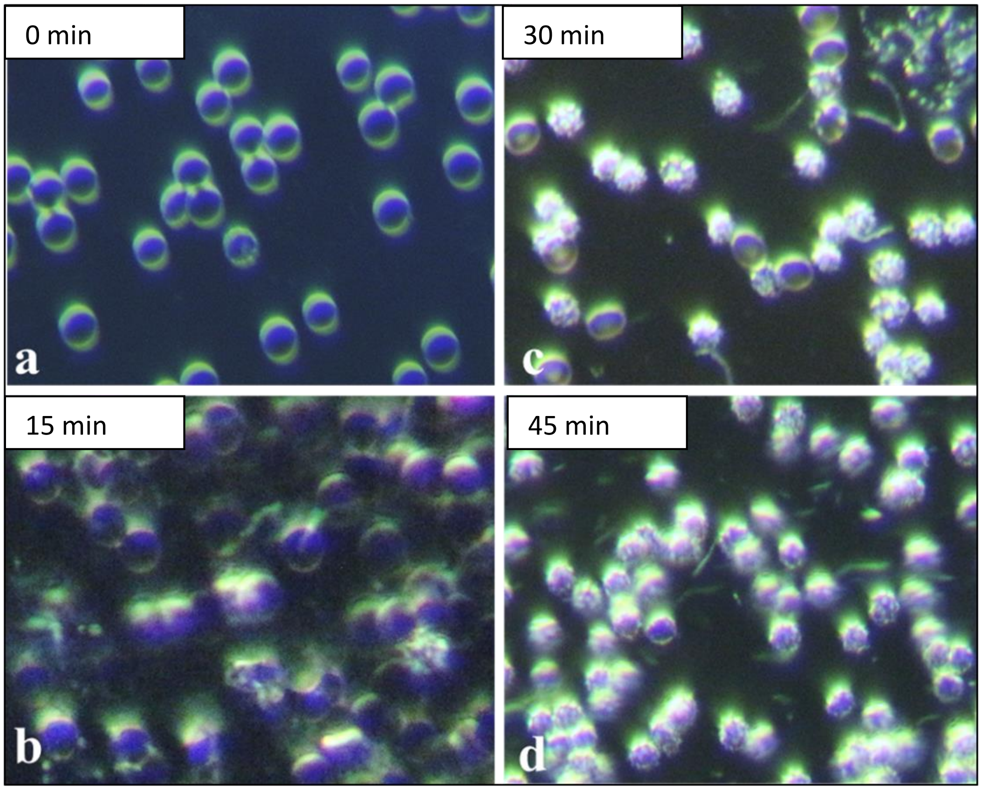

Figure S14. Dark field microscopy images showing time dependant live imaging of RBC's during lysis caused by phenylalanine fibrils.

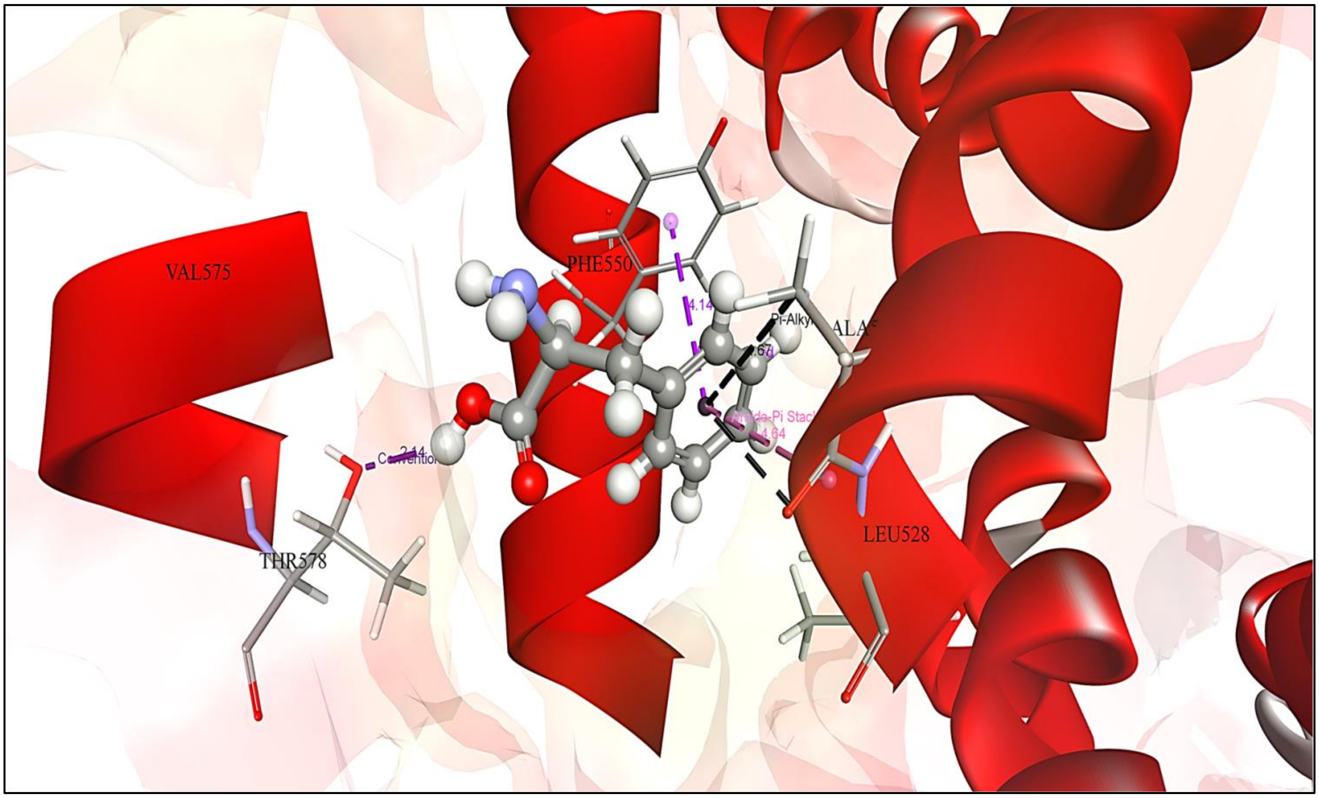

Sl. No.	Interacting residues	Bond Length (Å)	Interaction Type
**1**	PHE:H23 – A:THR578:OG1	2.14	Conventional hydrogen bond
**2**	A:PHE550 – PHE	4.14	Pi- Stacked
**3**	A:ALA527 C,O;LEU528:N–PHE	4.64	Amide Pi- Stacked
**4**	PHE – A:ALA527	4.67	Pi-Alkyl
**5**	PHE – A:LEU528	5.25	Pi-Alkyl

Figure S15. Summary of Bovine Serum Albumin PDB: 4F5S–Phenylalanine interaction. The value of – CDOCKER energy was 27.0621 kcal mol^{-1} and the value of – CDOCKER interaction energy was found to be 28.9882 kcal mol^{-1}.

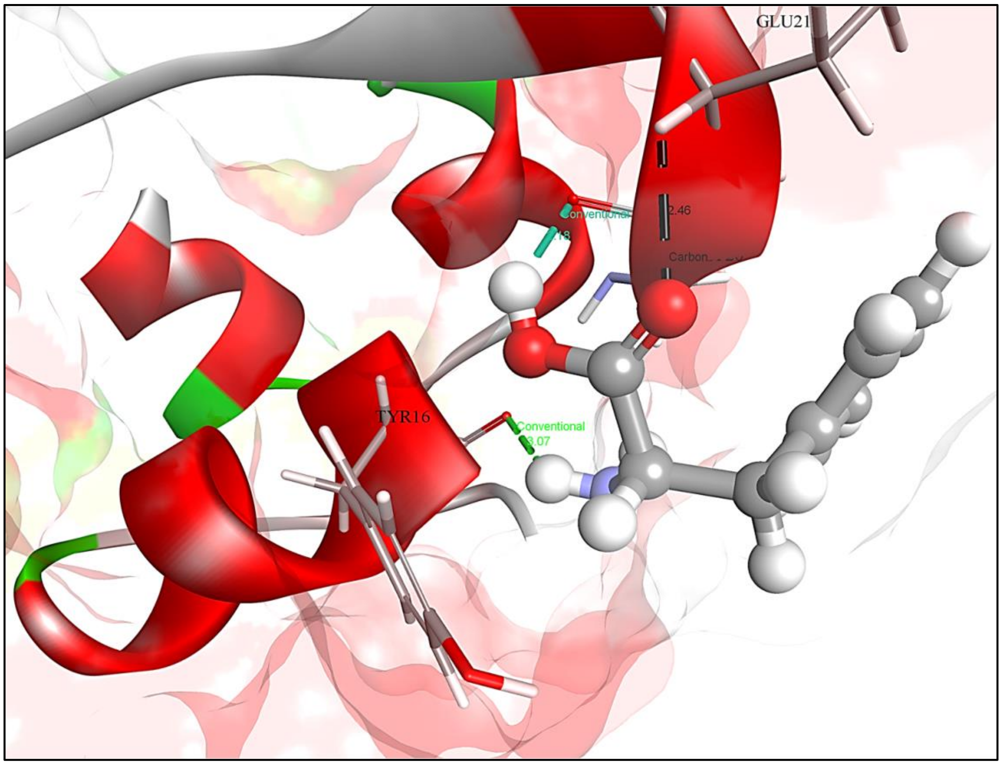

Sl. No.	Interacting residues	Bond Length (Å)	Interaction Type
**1**	B:GLU21:HA – PHE:O2	2.46	Carbon Hydrogen Bond
**2**	PHE:H23 – B:GLU20:O	2.18	Conventional hydrogen bond
**3**	PHE:H19 – B:TYR16:O	3.07	Conventional hydrogen bond

Figure S16. Summary of Insulin PDB 4I5Z–Phenylalanine interaction. The value of – CDOCKER energy was 14.3516 kcal mol^{-1} and the value of – CDOCKER interaction energy was found to be 14.3759 kcal mol^{-1}.

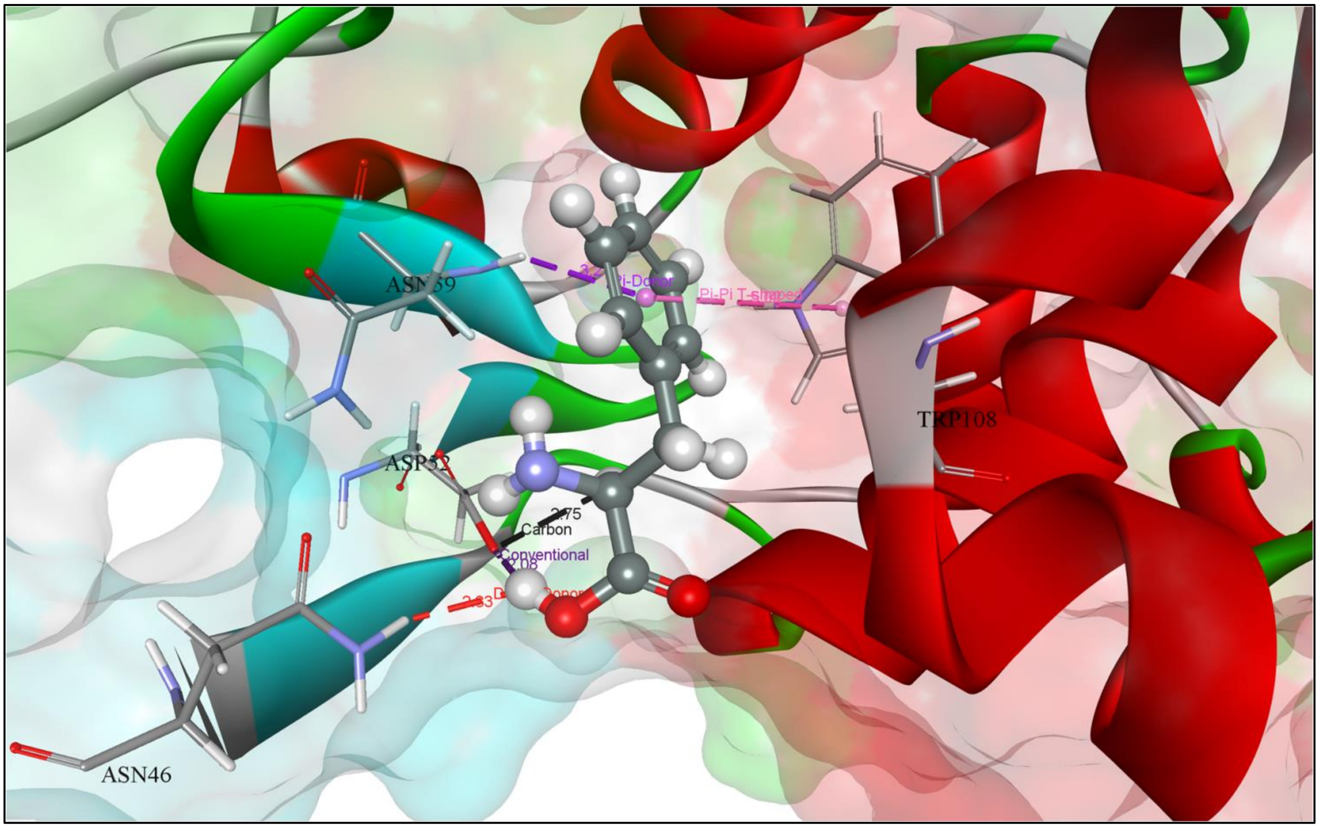

Sl. No.	Interacting residues	Bond Length (Å)	Interaction Type
**1**	PHE:H23 – A:ASP52:OD2	2.08	Conventional hydrogen bond
**2**	A:ASN46:HD22 – PHE:H23	2.33	Donar-Donar hydrogen bond
**3**	PHE:H15 – A:ASP52:OD2	2.75	Carbon hydrogen bond
**4**	A:ASN59:NH – PHE	3.21	Pi-Donar hydrogen bond
**5**	A:TRP108 – PHE	5.02	Pi-Pi T-shaped interaction

Figure S17. Summary of Lysozyme PDB 193L–Phenylalanine interaction. The value of – CDOCKER energy was 26.8447 kcal mol^{-1} and the value of – CDOCKER interaction energy was found to be 29.0785 kcal mol^{-1}.

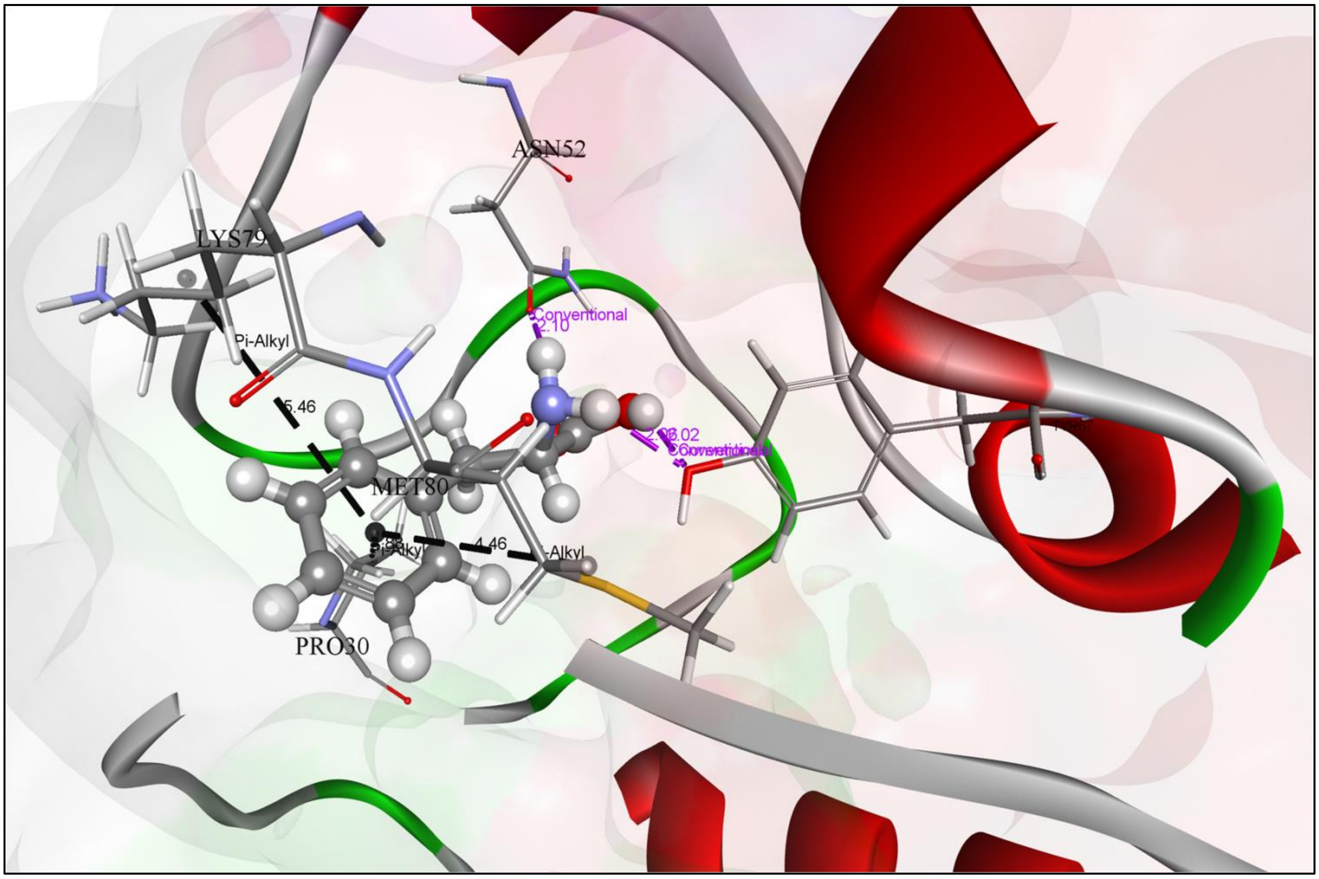

Sl. No.	Interacting residues	Bond Length (Å)	Interaction Type
**1**	PHE:H23 – A:TYR67:OH	2z.02	Conventional hydrogen bond
**2**	PHE:H18 – A:TYR67:OH	2.06	Conventional hydrogen bond
**3**	PHE:H19 – A:ASN52:OD1	2.10	Conventional hydrogen bond
**4**	PHE – A:MET80	4.46	Pi-Alkyl
**5**	PHE – A:PRO30	4.88	Pi-Alkyl
**6**	PHE – A:LYS79	5.46	Pi-Alkyl

Figure S18. Summary of Cytochrome C PDB: 1HRC–Phenylalanine interaction. The value of – CDOCKER energy was 26.4082 kcal mol^{-1} and the value of – CDOCKER interaction energy was found to be 28.6882 kcal mol^{-1}.

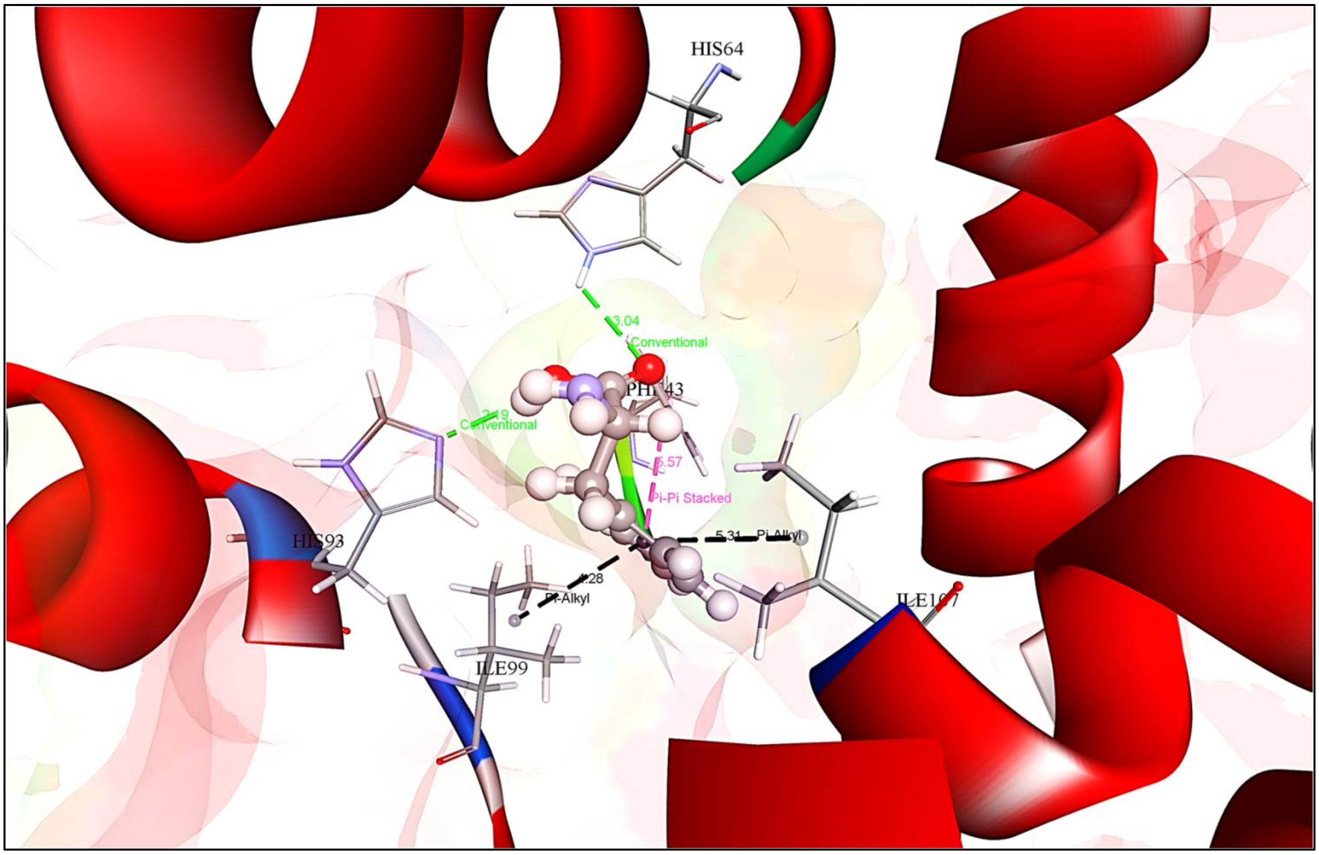

Sl. No.	Interacting residues	Bond Length (Å)	Interaction Type
**1**	PHE:H23 – A:HIS93:NE2	2.19	Conventional hydrogen bond
**2**	PHE:O2 – A:HIS64:HE2	3.04	Conventional hydrogen bond
**3**	PHE – A:ILE99	4.28	Pi-Alkyl
**4**	PHE – A:ILE107	5.31	Pi-Alkyl
**5**	PHE – A:PHE43	5.57	Pi-Pi stacking

Figure S19. Summary of Myoglobin PDB: 1DWR–Phenylalanine interaction. The value of – CDOCKER energy was 22.615 kcal mol^{-1} and the value of – CDOCKER interaction energy was found to be 24.6064 kcal mol^{-1}.

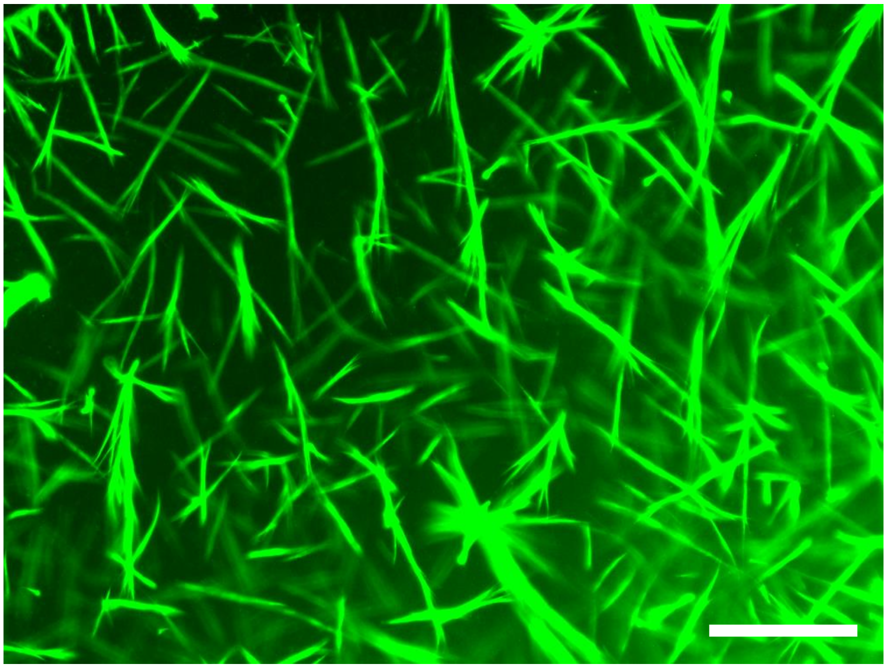

Figure S20. Fluorescent microscopic image (stained with an amyloid specific dye Thioflavin T) showing higher order structures generated from a phenylalanine-induced aggregation reaction of a soluble mixture of amino acids (in PBS at 37°C). Scale bar, 20μm.

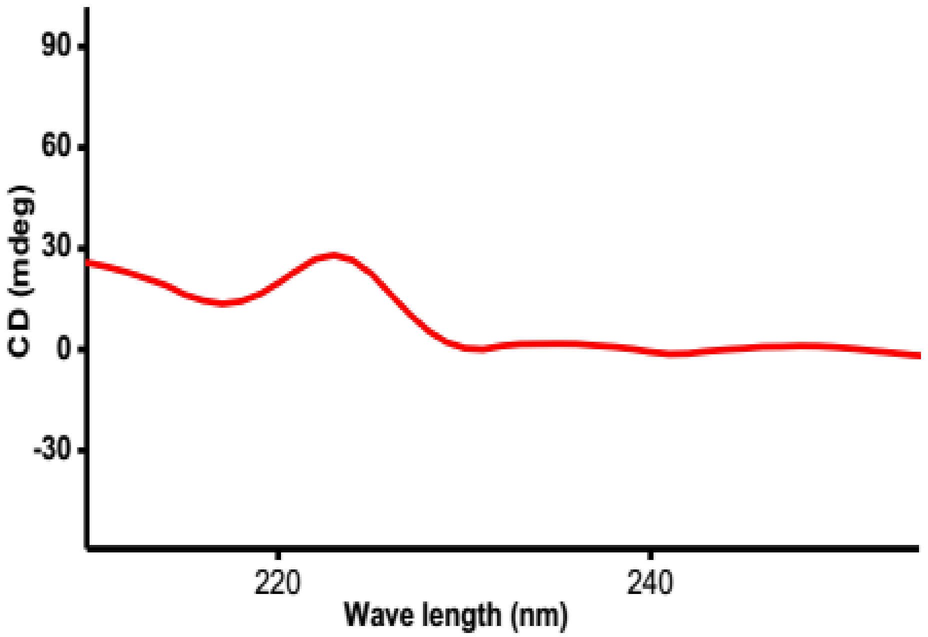

Figure S21. CD profile of phenylalanine aggregates in PBS. The concentration of the sample was ~ 1mM.

References:

1. Surtees, R. & Blau, N. The neurochemistry of phenylketonuria. *Eur. J. Pediatr*. **159**, 109–113 (2000).
2. de Groot, M. J., Hoeksma, M., Blau, N., Reijngoud, D. J.& Spronsen van, F. J. Pathogenesis of cognitive dysfunction in phenylketonuria: review of hypotheses. *Mol. Genet.Metab*. **99**, 86–89 (2010).
3. Burlina, A. B., Ferrari, B., Suppiej, L. V., Zacchello, F. & Burlina, A. P. Measurement of neurotransmitter metabolites in the cerebrospinal fluid of phenylketonuric patients under dietary treatment. *J. Inherit. Metab. Dis.* **23,** 313–316 (2000).
4. McKean, C. M. The effects of high phenylalanine concentrations on serotonin and catecholamine metabolism in the human brain. *Brain Res*. **47,** 469–476 (1972).
5. Sitta, A., Barschak, A. G., Deon, M., de Mari, J. F., Barden, A. T., Vanzin C. S., Biancini G. B., Schwartz I. V., Wajner, M. & Vargas, C. R. L-Carnitine Blood Levels and Oxidative Stress in Treated Phenylketonuric Patients. *Cell Mol Neurobiol*. **29**, 211-218 (2009).
6. Kleopatra, H., Schulpis, Kariyannis, C. & Papassotirioub, I. Serum levels of neural protein S-100B in phenylketonuria. *Clin Biochem*. **37,** 76-79 (2004).
7. Sherpa, D., Stigum, H., Chongsuvivatwong, V., Luobu, O., Thelle, D. S., Nafstad, P. & Bjertness, E. Lipid Profile and Its Association with Risk Factors for Coronary Heart Disease in the Highlanders of Lhasa. *High Alt Med Biol*. **12,** 57-63 (2011).

8. Schulpi, K. H. & Scarpalezou, A. Triglycerides, cholesterol HDL, LDL, and VLDL cholesterol in serum of phenylketonuric children under dietary control. *Clin Pediatr* **28**, 466–469 (1989).
9. Kleopatra, H. & Schulpis, E. D. Plasma Leptin Concentrations in Phenylketonuric Patients. *Horm Res*. **53**, 32-35 (2000).
10. Sitta, A., Manfredinic, V., Biasi, L., Treméad, R., Schwartzb, V. D., Wajnera, M. & Vargas,C. R. Evidence that DNA damage is associated to phenylalanine blood levels in leukocytes from phenylketonuric patients. *Mutation Research* **679**, 13–16 (2009).
11. Tsakirisa, S., Schulpisb, K. H., Tjamouranisb, J., Michelakakisb, H. & Karikasc, G. A. Reduced acetylcholinesterase activity in erythrocyte membranes from patients with phenylketonuria. *Clinical Biochemistry* **35,** 615–619 (2002).
12. Schulpisa, K. H., Tjamouranisa, J., Karikasb, G. A., Michelakakisa, H. & Tsakirisc, S. In vivo effects of high phenylalanine blood levels on Na,K-ATPase, Mg2-ATPase activities and biogenic amine concentrations in phenylketonuria. *Clinical Biochemistry* **35**, 281–285 (2002).
13. Artuch, R., Colome, C.& Vilaseca MA, et al. Plasma phenylalanine is associated with decreased serum ubiquinone-10 concentrations in phenylketonuria. *J Inher Metab Dis*. **24**, 359–66 (2001).
14. Infante, I. P. & Huszagh,V. A. Impaired Arachidonic (20:4n-6) and Docosahexaenoic (22:6n-3) Acid Synthesis by Phenylalanine Metabolites as Etiological Factors in the Neuropathology of Phenylketonuria. *Molecular Genetics and Metabolism* **72**, 185–198 (2001).
15. Ruppert, E.S. Hemolytic disease in association with elevation of serum phenylalanine. *The journal of pediatrics* **71,** 152 (1987).
16. Tenidis, K., Waldner, M., Bernhagen, J., Fischle, W., Bergmann, M., Weber, M., Merkle, M.L., Voelter, W., Brunner, H. & Kapurniotu A. Identification of a penta- and hexapeptide of islet amyloid polypeptide (IAPP) with amyloidogenic and cytotoxic properties. *J Mol Biol.* **295**, 1055-1071 (2000).
17. Tjernberg, L. O., Na¨slund, J., Lindqvist, F., Johansson, J., Karlstro¨m, A. R., Thyberg, J., Terenius, L. & Nordstedt, C. Controlling Amyloid β-Peptide Fibril Formation with Protease-stable Ligands. *J. Biol. Chem.* **271,** 8545–8548 (1996).
18. Findeis, M. A., Musso, G. M., Arico-Muendel, C. C., Benjamin, H. W., Hundal, A. M., Lee, J. J., Chin, J., Kelley, M., Wakefield, J., Hayward, N. J. & Molineaux, S. M. Modified-peptide inhibitors of amyloid beta-peptide polymerization. *Biochemistry* **38,** 6791–6800 (1999).
19. Pallitto, M. M., Ghanta, J., Heinzelman, P., Kiessling, L. L. & Murphy, R. M. Recognition sequence design for peptidyl modulators of beta-amyloid aggregation and toxicity. *Biochemistry* **38,** 3570–3578 (1999).
20. Soto, C., Sigurdsson, E. M., Morelli, L., Kumar, R. A., Castano, E. M. & Frangione, B. β-sheet breaker peptides inhibit fibrillogenesis in a rat brain model of amyloidosis: Implications for Alzheimer's therapy. *Nat. Med.* **4,** 822–826 (1998).
21. Ha¨ggqvist, B., Na¨slund, J., Sletten, K., Westermark, G. T., Mucchiano, G., Tjernberg, L. O., Nordstedt, C., Engstro¨m, U. & Westermark, P. Medin: An integral fragment of aortic smooth

muscle cell-produced lactadherin forms the most common human amyloid. *Proc. Natl. Acad. Sci.* **96,** 8669–8674 (1999).

22. Maury, C. P. & Nurmiaho-Lassila, E. L. A possible role for -stacking in the self-assembly of amyloid fibrils. *Biochem. Biophys. Res. Commun.* **183,** 227–231 (1992).
23. Westermark, G. T., Engstro¨m, U. & Westermark, P. The N-terminal segment of protein AA determines its fibrillogenic property. *Biochem. Biophys. Res. Commun.* **182,** 27–33 (1992).
24. Reches, M., Porat, Y., & Gazit E. Amyloid fibril formation by pentapeptide and tetrapeptide fragments of human calcitonin. *J Biol Chem* **277**, 35475-80 (2002).
25. Bemporad, F., Taddei, N., Stefani, M. & Chiti, F. Assessing the role of aromatic residues in the amyloid aggregation of human muscle acylphosphatase. *Protein Science* **15**, 862–870 (2006).
26. Kedar, I., Ravid, M., & Sohar, E. In vitro synthesis of 'amyloid' fibrils from insulin, calcitonin-and parathormone. *Israel J. Med. Sci.* **12**, 1137 (1976).
27. Benvenga, S., Trimarchi, F., & Facchiano, A. Homology of calcitonin with the amyloid-related proteins. *J. Endocrinol. Invest*. **17**, 119–122 (1994).
28. Berger, G., Berger, N., Guillaud, M. H., Trouillas, J., & Vauzelle, J. L. Calcitonin-like immunoreactivity of amyloid fibrils in medullary thyroid carcinomas. An immunoelectron microscope study. *Arch. A Pathol. Anat. Histopathol*. **412**, 543–551 (1988).
29. Vidal, R., Frangione, B., Rostagno, A., Mead, S., Revesz, T., Plant, G., & Ghiso, J. A stop-codon mutation in the BRI gene associated with familial British dementia. *Nature* **399**, 776–781 (1999).
30. Jones, S., Manning, J., Kad, N. M. & Radford, S. E. Amyloid-forming peptides from beta2-microglobulin – insights into the mechanism of fibril formation in vitro. *J Mol Biol* **325**, 249–257 (2003).
31. Prusiner, S. B., Scott, M. R., DeArmond, S. J. & Cohen, F. E. Prion Protein Biology. *Cell.* **93**,337–348 (1998).
32. Priola, S. A. & Chesebro, B. J. Abnormal properties of prion protein with insertional in different cell types. *Biol. Chem.* **273**, 11980–11985 (1998).
33. Bessis, M. *Corpuscles Atlas of Red Blood Cell Shapes.* Springer ISBN: 978-3-642-65659-0 (1974).
34. Bessis, M. *Blood Smears Reinterpreted*. Springer ISBN 978-3-642-66094-8 (1977).
35. Bevinahalli, N. N., Rajalakshmi, T. & Shubha, B. Cutaneous amyloidosis at the site of insulin injection with coexistence of acanthosis nigricans. *Indian Journal of Pathology and Microbiology* **57**, 127-129 (2014).
36. Nagase, T. *et al.* Insulin-derived Amyloidosis and Poor Glycemic Control: A Case Series. *The American Journal of Medicine* **127**, 450-454 (2014).
37. Kopf, D., Muhlen, I., Kroning, G., Sendzik, I., Huschke, B. & Lehnert, H. Insulin sensitivity and sodium excretion in normotensive offspring and hypertensive patients. *Metabolism* **50**, 929-935 (2001).
38. Burnol, A. F., Morzyglod, L. & Popineau, L. Cross-talk between insulin signaling and cell proliferation pathways. *Ann Endocrinol* **74**, 74-78 (2013).

39. Nilsen, T. I. & Vatten, L.J. Prospective study of colorectal cancer risk and physical activity, diabetes, blood glucose and BMI: exploring the hyperinsulinaemia hypothesis. *Br J Cancer* **84**, 417-22 (2001).
40. Després, J.P. *et al.* Hyperinsulinemia as an independent risk factor for ischemic heart disease. *N Engl J Med*. **334,** 952-957 (1996).
41. Chu, N., Spiegelman, D., Hotamisligil, G. S., Rifai, N., Stampler, M. & Rimm, E. B. Plasma insulin, leptin, and soluble TNF receptors levels in relation to obesity-related atherogenic and thrombogenic cardiovascular disease risk factors among men. *Atherosclerosis* **157,** 495-503 (2001).
42. Cersosimo, E. & DeFronzo, R. A. Insulin resistance and endothelial dysfunction: the road map to cardiovascular diseases. *Diabetes Metab Res Rev*. **22**,423-436 (2006).
43. Eschwège, E. The dysmetabolic syndrome, insulin resistance and increased cardiovascular (CV) morbidity and mortality in type 2 diabetes: aetiological factors in the development of CV complications. *Diabetes Metab*. **65**, 19-27 (2003).
44. Hammarsten, J. & Hogstedt, B. Hyperinsulinaemia as a risk factor for developing benign prostatic hyperplasia. *Eur Urol*. **39,** 151-158 (2001).
45. Hansen, J. *et al.* Plasma follistatin is elevated in patients with type 2 diabetes: relationship to hyperglycemia, hyperinsulinemia, and systemic low-grade inflammation. *Diabetes Metab Res Rev*. **29**, 463-472 (2013).
46. Valleix, S., Drunat, S., Philit, J. B., Adoue, D., Piette, J. C., Droz, D., MacGregor, B., Canet, D., Delpech, M. & Grateau, G. Hereditary renal amyloidosis caused by a new variant lysozyme W64R in a French family. *Kidney Int.* **61**, 907–912 (2002).
47. Sandberg-Gertzen, H., Ericzon, B. G. & Blomberg, B. Primary amyloidosis with spontaneaous splenic rupture, cholestasis, and liver failure with emergency liver transplantation. *Am J Gastroenteraol.* **93**, 2254–2256 (1998).
48. Harrison, R. F., Hawkins, P. N., Roche, W. R., MacMahon, R. F., Hubscher, S. G. & Buckels, J. A. 'Fragile' liver and massive hepatic haemorrhage due to hereditary amyloidosis. *Gut.* **38**, 151–152 (1996).
49. Hendgen-Cotta, U. B., Flögel, U., Kelm, M. & Rassafl, T. Unmasking the Janus face of myoglobin in health and disease. *The Journal of Experimental Biology* **213**, 2734-2740 (2010).
50. Romero-Herrera, A. E., Lehmann, H., Tomlinson, B. E. & Walto, J. N. Myoglobin in Primary Muscular Disease I. Duchenne Muscular Dystrophy and II. Muscular Dystrophy of Distal Type. *Journal of Medical Genetics* **10**, 309-322 (1973).
51. Hashimoto, M., Takeda, A., Hsu, L. J., Takenouchi, T., & Masliah, E. Role of Cytochrome c as a Stimulator of a-Synuclein Aggregation in Lewy Body Disease. *The Journal of Biological Chemistry* **274**, 28849–28852 (1999).
52. MacKay, D. & Miller, A. L. Nutritional Support for Wound Healing. *Alternative Medicine Review* **8,** 359-342 (2003).
53. Guyton, A. Human Physiology and Mechanisms of Disease. *Philadelphia: WB Saunders Company* 126–130 (1992).
54. Melmed, S., Polonsky, K. S., Larsen, P. R. & Kronenberg, H. M. *Williams Textbook of Endocrinology*. Elsevier Saunders. 12th ed. (2011).
55. Hankiewicz, J. & Swierczek, E. Lysozyme in human body fluids. *Clinica Chimica Acta* **57,** 205-209 (1974).

56. Garcia-Martinez, R., Caraceni, P., Bernardi, M., Gines, P., Arroyo, V. & Jalan, R. Albumin: Pathophysiologic Basis of Its Role in the Treatment of Cirrhosis and Its Complications. *Hepatology*, **58**, 1836-1846 (2013).
57. Doweiko, J. P. & Nompleggi, D. J. The role o albumin in human physiology and pathophysiology, Part III: Albumin and disease states. *JPEN J Parenter Enteral Nutr* ***15**, 476-483 (1990).*
58. Louis-Jeune, C., Andrade-Navarro, M. A. & Perez-Iratxeta, C. Prediction of protein secondary structure from circular dichroism using theoretically derived spectra. *Protein: Structure, Function, and Bioinformatics*. **80**, 374-381 (2012).
